# Systems Pharmacology and Verification of ShenFuHuang Formula in Zebrafish Model Reveal Multi-Scale Treatment Strategy for Septic Syndrome in COVID-19

**DOI:** 10.3389/fphar.2020.584057

**Published:** 2020-09-15

**Authors:** Tengwen Liu, Yuhong Guo, Jingxia Zhao, Shasha He, Yunjing Bai, Ning Wang, Yan Lin, Qingquan Liu, Xiaolong Xu

**Affiliations:** ^1^ School of Chinese Medicine, Hong Kong Baptist University, Hong Kong, Hong Kong; ^2^ Beijing Institute of Traditional Chinese Medicine, Beijing Hospital of Traditional Chinese Medicine, Capital Medical University, Beijing, China; ^3^ Beijing Key Laboratory of Basic Research with Traditional Chinese Medicine on Infectious Diseases, Beijing, China

**Keywords:** COVID-19, sepsis, traditional Chinese medicine, systems pharmacology, zebrafish

## Abstract

The outbreak of coronavirus disease 2019 (COVID-19) has affected millions of people worldwide. Critically ill COVID-19 patients develop viral septic syndrome, including inflammatory damage, immune dysfunction, and coagulation disorder. In this study, we investigated ShenFuHuang formula (SFH), a traditional Chinese medicine, which has been widely used as complementary therapy for clinical treatment of COVID-19 in Wuhan, to understand its pharmacological properties. Results of systems pharmacology identified 49 active compounds of SFH and their 69 potential targets, including GSK3β, ESR1, PPARG, PTGS2, AKR1B10, and MAPK14. Network analysis illustrated that the targets of SFH may be involved in viral disease, bacterial infection/mycosis, and metabolic disease. Moreover, signaling pathway analysis showed that Toll-like receptors, MAPK, PPAR, VEGF, NOD-like receptor, and NF-kappa B signaling pathways are highly connected with the potential targets of SFH. We further employed multiple zebrafish models to confirm the pharmacological effects of SFH. Results showed that SFH treatment significantly inhibited the inflammatory damage by reducing the generation of neutrophils in Poly (I:C)-induced viral infection model. Moreover, SFH treatment could improve the phagocytosis of macrophages and enhance the expression of immune genes in an immune deficiency model. Furthermore, SFH treatment exhibited promising anti-thrombosis effect in a thrombus model. This study provided additional evidence of SFH formula for treating COVID-19 patients with septic syndrome using multiple-scale estimation.

## Introduction

The outbreak of coronavirus disease 2019 (COVID-19) pandemic caused by the novel severe acute respiratory syndrome coronavirus 2 (SARS-CoV-2, 2019‐nCoV) has caused an enormous impact worldwide since the end of 2019, resulting in great loss to global health and economy (Wu et al., 2020). It has been confirmed that bats and birds are the hosts of typical coronavirus, with zoonotic spread and animal-animal-human transmission (Connors and Levy, 2020). By July 8, 2020, data from the World Health organization (WHO) showed that the number of COVID-19 confirmed cases worldwide had increased to over 11 000 000. Although most COVID-19 patients suffered from a mild illness, 5% of the patients developed severe lung injury or even systemic organ failure. The number of confirmed deaths worldwide has reached 500 000. Hence, it is critical to urgently improve the therapeutic schedule and develop more effective drugs against SARS-CoV-2. The efforts of clinicians worldwide have led to considerable experience and understanding of this infectious disease. Recent studies reported that many critically ill COVID-19 patients developed typical septic syndrome, including inflammatory injury, immune dysfunction, coagulation disorder, and multiple organ failure (Bellinvia et al., 2020; Coronado et al., 2020; Li et al., 2020). Diagnosis and clinical symptoms of these patients met the criteria of the Sepsis-3 International Consensus. Guidelines on the management of critically ill adults with COVID-19 established by Surviving Sepsis Campaign (SSC) also emphasized the treatment and supportive care of patients with septic syndrome (Alhazzani et al., 2020).

Infectious complications in critically ill patients are known to activate multiple systemic coagulation and inflammatory responses that are vital for host defense. Early inflammatory response and activation of coagulation system are critical for the elimination of SARS-CoV-2 *in vivo (* Giannis et al., 2020; Merad and Martin, 2020; Tay et al., 2020). However, when the inflammatory reaction is not controlled in time, the exogenous coagulation pathway will promote coagulation reaction. The uncontrolled coagulation dysfunction may lead to vascular damage and eventually disseminated intravascular coagulation (DIC) (Jose and Manuel, 2020). Given the important role of the inflammation and coagulation system in the development of sepsis, it is critical to identify drugs that can effectively modulate these pathological processes. Early use of anti-infective drugs combined with hemodynamic support achieves better therapeutic effect, but when the immune balance of the host is disrupted, the interaction between inflammatory response and coagulation function complicates the treatment.

ShenFuHuang (SFH) formula, which is composed of *Panax ginseng C.A.Mey*, *Aconitum carmichaeli Debeaux*, and *Rheum palmatum L.*, has been extensively used for clinical treatment of critical illnesses, including sepsis and septic shock. According to our statistics, SFH formula was empirically applicated on more than 500 critically ill patients with sepsis or septic shock in three hospitals during the COVID-19 outbreak in Wuhan. However, as a traditional Chinese medicine (TCM) with complex composition, it is necessary to understand its pharmacological effects and mechanisms *via* multi-scale investigations. Systems pharmacology has been used to identify the active compounds and potential targets of herbal medicines, combining pharmaco-chemistry information and multi-target prediction with network analyses. In this study, we employed systems pharmacology to reveal the underlying mechanisms of SFH. Zebrafish (*Danio rerio*) have become powerful tools in drug development research due to their molecular conservation, genetic accountability, ease of experimental use, and diverse behavioral properties. Zebrafish models and tests are particularly useful in genetics research, drug screening, global inflammatory research, as well as coagulation system disorders.

In this study, we used multiple tools to understand the effects and molecular mechanisms of SFH, in order to provide new insights into the clinical application of TCM for treating COVID-19 patients with septic syndrome.

## Materials and Methods

### Preparation of SFH Formula

SFH formula, consists of Hong Shen (*Panax ginseng C.A.Mey*) (ratio 1/4), Fu Zi (*Aconitum carmichaeli Debeaux*) (ratio 2/4), and Da Huang (*Rheum palmatum L*.) (ratio 1/4), was decocted and provided by TCM Pharmacy of Beijing hospital of traditional Chinese Medicine. Briefly, 120 g crude drugs of SFH formula were soaked and decocted in 400 ml pure water for 30 min. Then the water decoction was concentrated to 120 ml, and the final dosage of crude drugs was 1 g/ml. The dosages of SFH formula used for this study were 1.11, 3.33, and 10 μl/ml water (autoclaved and sterilized), respectively.

### Reagents and Herbs

Indomethacin (No. 13931) and arachidonic acid (No. A1831030) were purchased from Aladdin Biochemical Technology Co., Ltd (Shanghai, China). LPS (No. 017M4112V) and aspirin (No. MKCD0957) were purchased from Sigma-Aldrich Chemical (St. Louis, MO, USA). FastQuant RT Kit (No. KR106) was purchased from TIANGEN Biotech Co., Ltd (Beijing, China). TRIzol^®^ reagent was purchased from Gibco (Grand Island, NY, USA). *Panax ginseng C.A.Mey* was purchased from Beijing TaiYangShuKang herbs company (Beijing, China, Voucher number 1805060). *Aconitum carmichaeli Debeaux* was purchased from China SinoPharm (Beijing, China, Voucher number xf5271). *Rheum palmatum L*. was purchased from Beijing Xinglin pharmaceutical (Beijing, China, Voucher number 20012201)

### Data Set Construction

The current data were obtained from literature mining and the TCM pharmacology analysis platforms, including TCMSP (http://lsp.nwsuaf.edu.cn/tcmsp.php) (Ru et al., 2014) and ETCM (http://www.tcmip.cn/ETCM/) (Xu et al., 2019). We collected the physical and chemical information of 231 compounds from SFH formula, including 92 compounds of *Rheum palmatum L.*, 74 compounds of *Panax ginseng C.A.Mey*, and 65 compounds of *Aconitum carmichaeli Debeaux*.

### Active Compound Screening Model

#### Oral Bioavailability

Oral Bioavailability (OB) is an important pharmacokinetic parameter in the drug’s ADME (absorption, distribution, metabolism, excretion) curve. In this study, OB was evaluated based on OBioavail 1.1 (Xu et al., 2012) and IntegrOB. Suitable molecules with OB ≥ 30% are used as candidate compounds for further study.

#### Caco-2 Permeability

The oral absorption of drugs is mainly done by intestinal epithelial cells (IEC). In this study, the computer Caco-2 permeability prediction model was used to predict the intestinal permeability of all TCM components in TCMSP. Molecules with Caco-2 > −0.4 are considered to exhibit sufficient intestinal epithelial permeability.

#### Drug-Likeness

Drug similarity is a qualitative concept used in drug design to illustrate the “drug -Likeness (DL)” of a substance in terms of bioavailability and other factors. In this work, Tanimoto coefficient is used for estimating DL. Compounds with DL ≥ 0.18 are considered to have high drug-like properties and are selected as candidates for further study.

#### Half-Life

The half-life (HL) of a drug reflects the rate of elimination (excretion, biotransformation, storage, etc.) of the drug in the body, and represents the relationship between the time and drug in the body. The compounds with HL ≥ 4 in SFH were screened as candidate active molecules for study.

#### Drug Targeting

To obtain targets we used weighted ensemble similarity (WES) model, a comprehensive drug targeting method that integrates chemical, genomics, and pharmacological information to predict direct target data of SFH. Full data mining is performed in the TCMSP analysis platform to obtain the three-dimensional structure information corresponding to the molecule. Run the WES model with the obtained molecular three-dimensional structure as an index to obtain all species targets. Finally, put the targets from different sources in the UniProt (http://www.uniprot.org) database with a unified name, and then submit them to the PharmGKB database, Therapeutic Targets Database (TTD) and the Comparative Toxicogenomics Database (CTD) to delete redundant and wrong targets to ensure the accuracy of the targets (Zheng et al., 2015).

### Network Construction

We performed gene ontology functional enrichment analysis by using BatchQuery CTD database (http://ctdbase.org/tools/batchQuery.go) to obtain all disease information related to the targets. The drug-target network is constructed using the software Cytoscape 2.8.1. In the generated network, nodes represent compounds, proteins, or diseases, and edges represent compound-target, target-disease, and target-pathway interactions (Smoot et al., 2011).

### Ultra-Performance Liquid Chromatography-Tandem Mass Spectrometry (UPLC–MS/MS) Analysis

A Waters UPLC-MS/MS spectrometer equipped with a HESI-II probe was used to identify the main composition of SFH formula. The details of the protocol were as previously described (Xu et al., 2018). Data were collected and analyzed by using the Waters Masslynx 4.1 system.

### Zebrafish Models

#### Zebrafish Embryo and Larvae Maintenance

Wild type AB zebrafish, transgenic neutrophil fluorescent AB zebrafish, melanin allele mutated translucent Albino strain zebrafish were purchased from Hunter Biotech (Hangzhou, China). Zebrafish were maintained and raised according to the protocol described before (Yang et al., 2014; Mehrdana et al., 2017).

#### Poly I:C-Induced Viral Infection Model

Randomly select 180 transgenic neutrophil fluorescent AB zebrafish (3 days post fertilization, 3 dpf) into six-well plates. Each well (3 ml) contains 30 tails of zebrafish. Poly (I:C) (100 ng/fish) was injected into the swim bladder to establish a zebrafish infection model. The model zebrafish were administered with or without SFH formula at different dosages for 3 h. The concentration of the positive control indomethacin was 60 µM. After treatment with Poly (I:C) for 3 h, the zebrafish were collected for research.

#### Macrophage Activation Model

Randomly select 180 wild-type AB zebrafish (3 dpf) into a six-well plate. Each well contains 30 tails. Fish were given intravenous injection of Indian ink (10 nl/fish) to establish a model of phagocytosis of zebrafish macrophages. Neutral red was used to stain macrophage. The model zebrafish were administered with or without SFH formula at different dosages for 3 h for research.

#### AA-Induced Thrombosis Model

Randomly select 180 melanin allele mutated translucent Albino strain zebrafish (3 dpf) into a six-well plate. Zebrafish were given arachidonic acid to establish thrombosis model and then were administered with or without SFH formula at different dosages for 3 h for research. To evaluate the antithrombosis capacity, zebrafish was stained with o-anisidine staining solution, and the antithrombotic activity of medicines were quantitatively analyzed by calculating the staining intensity of zebrafish heart erythrocytes (Zhu et al., 2016).

Efficacy(%)=[S(drug)-S(model)]/[S(vehicle)-S(model)]×100%

### Staining

The zebrafish was fixed with 4% paraformaldehyde. After fixation, the zebrafish was transferred to 70% ethanol for dehydration, embedding, sectioning, staining, and mounting. The stained zebrafish sections were analyzed for pathology.

### Real-Time PCR

After extracting total zebrafish RNA from each experimental group using the classic Trizol method, the concentration and purity of total RNA were determined using Thermo ultra-micro spectrophotometer. The RNA was then quantified by UV spectrophotometry (Thermo, NanoDrop 2000). Then use the PrimeScript^®^ RT kit to amplify the transcribed cDNA according to the manufacturer’s instructions. Perform real-time quantitative PCR according to the manufacturer’s instructions (SYBR Green PCR Reagent kit). The primer sequences used are listed in **Supplementary Table S1**.

### Statistical Analysis

SPSS15.0 software was used to analyze the data. The analysis of variance was combined with Dunnett’s T-test for statistical analysis. p <0.05 indicated a significant difference.

## Results

In this study, parameters including OB (≥ 30%), DL (≥ 0.18), Caco-2 (> −0.4), and HL (≥ 4) were utilized to identify the active compounds of SFH. Compounds with properties OB < 30%, DL < 0.18, Caco-2 ≤ −0.4, and HL< 4 were considered as candidate compounds, since there are some typical pharmacodynamic molecules that possess well-documented biological activities established in *in-vivo* and *in-vitro* studies. Finally, 49 potential ingredients were screened as candidate compounds of SFH (**Table 1**).

**Table 1:** Candidate Information.

Molecular ID	Compound	Herb	OB	Caco2	DL	HL	Structure
M1	beta-sitosterol	*Rheum palmatum* L., Panax ginseng C.A.Mey	36.91	1.32	0.75	5.35	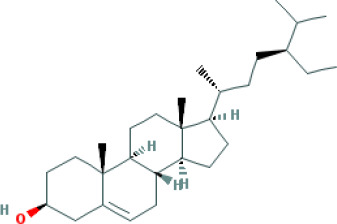
M2	sitosterol	*Aconitum carmichaeli* Debeaux	36.91	1.32	0.75	5.37	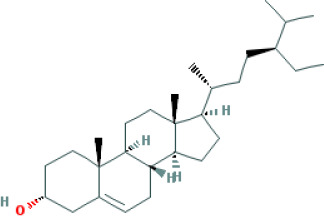
M3	aloe-emodin	*Rheum palmatum* L.	83.38	-0.12	0.24	31.5	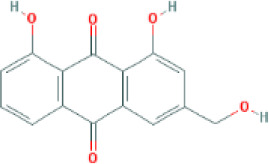
M4	emodin	*Rheum palmatum* L.	24.40	0.22	0.24	0	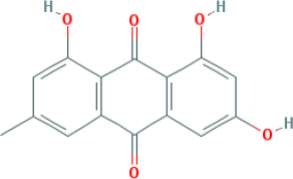
M5	chrysophanol	*Rheum palmatum* L.	18.64	0.62	0.21	0	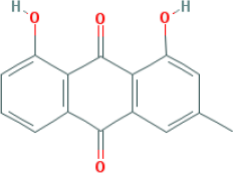
M6	dioctyl phthalate	*Panax ginseng* C.A.Mey	40.58	0.95	0.40	9.73	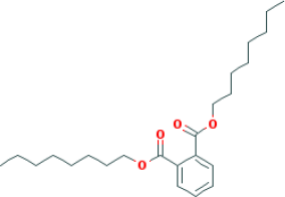
M7	11,14-eicosadienoic acid	*Aconitum carmichaeli* Debeaux	39.99	1.22	0.20	5.60	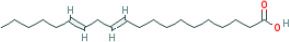
M8	eupatin	*Rheum palmatum* L.	50.80	0.53	0.41	13.9	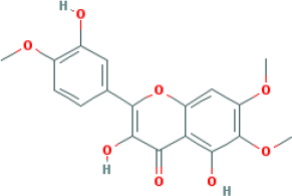
M9	chrysophanol glucoside	*Rheum palmatum* L.	20.06	-1.17	0.76	0	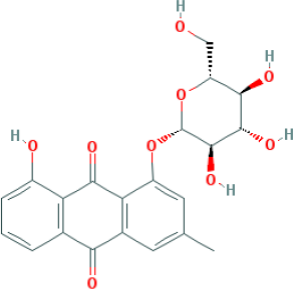
M10	rhein	*Rheum palmatum* L.	47.07	-0.20	0.28	32.1	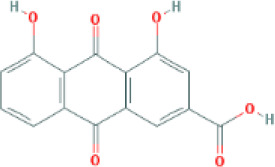
M11	daucosterol_qt	*Rheum palmatum* L.	35.89	1.35	0.70	6.11	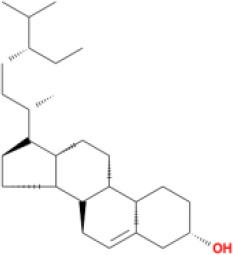
M12	Deltoin	*Aconitum carmichaeli* Debeaux	46.69	0.55	0.37	7.69	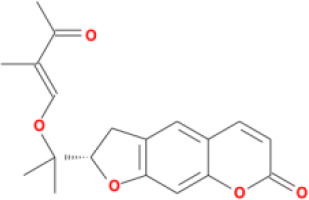
M13	karanjin	*Aconitum carmichaeli* Debeaux	69.56	1.22	0.34	13.1	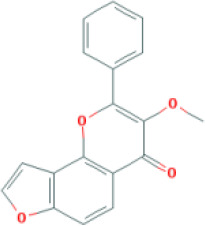
M14	fuzitine	*Aconitum carmichaeli* Debeaux	25.78	1.02	0.54	0	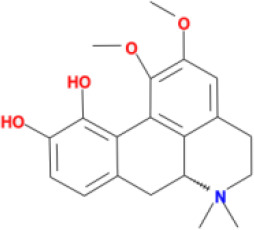
M15	carnosifloside I_qt	*Aconitum carmichaeli* Debeaux	38.15	0.28	0.79	6.99	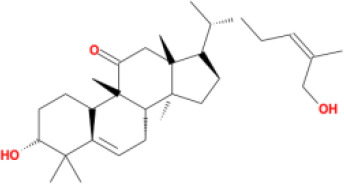
M16	ginsenoside rh2	*Panax ginseng* C.A.Mey	36.32	-0.50	0.56	11.07	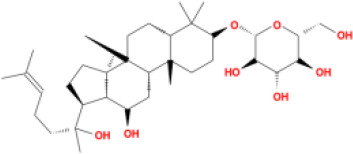
M17	ginsenoside rf	*Panax ginseng* C.A.Mey	17.74	-2.23	0.24	0	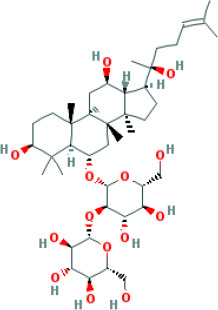
M18	ginsenoside R0_qt	*Panax ginseng* C.A.Mey	17.41	0.43	0.76	0	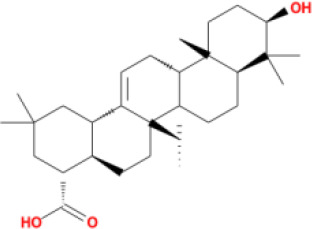
M19	mesaconitine	*Aconitum carmichaeli* Debeaux	8.70	-0.35	0.25	0	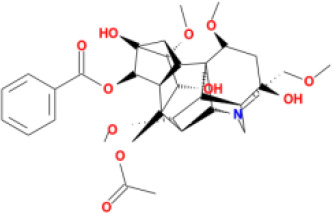
M20	(6Z,10E,14E,18E)-2,6,10,15,19,23-hexamethyltetracosa-2,6,10,14,18,22-hexaene	*Panax ginseng* C.A.Mey	33.55	2.07	0.42	3.14	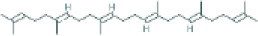
M21	ginsenoside-Rh1	*Panax ginseng* C.A.Mey	3.86	-1.17	0.57	0	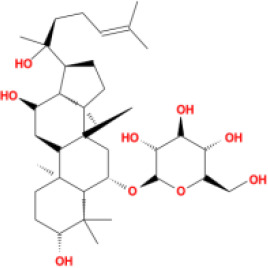
M22	20(R)-ginsenoside Rg2	*Panax ginseng* C.A.Mey	10.09	-1.96	0.26	0	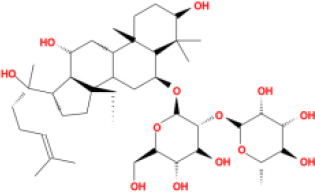
M23	ginsenoside Rb1	*Panax ginseng* C.A.Mey	6.24	-3.99	0.04	0	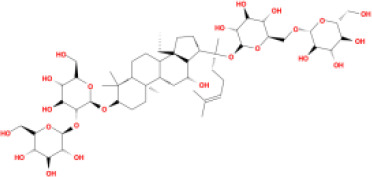
M24	ginsenoside-Rb2	*Panax ginseng* C.A.Mey	6.02	-3.92	0.04	0	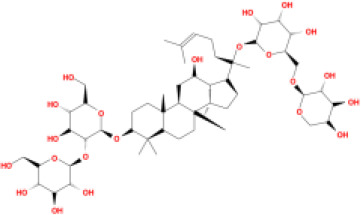
M25	ginsenoside-Rc	*Panax ginseng* C.A.Mey	8.16	-3.97	0.04	0	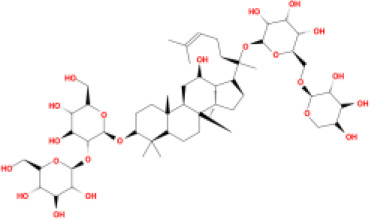
M26	ginsenoside Re	*Panax ginseng* C.A.Mey	4.27	-3.20	0.12	0	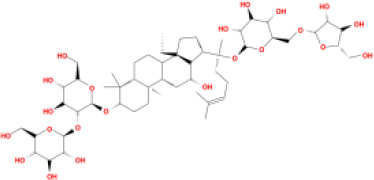
M27	notoginsenoside R2	*Panax ginseng* C.A.Mey	17.74	-2.22	0.28	0	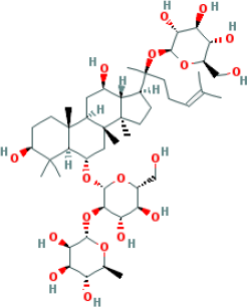
M28	ginsenoside Rg2_qt	*Panax ginseng* C.A.Mey	20.12	0.05	0.82	0	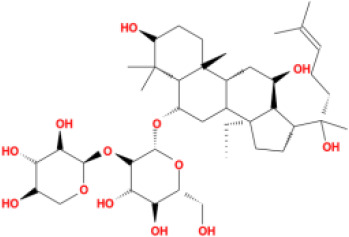
M29	ginsenoside Rg2_qt	*Panax ginseng* C.A.Mey	20.12	0.05	0.82	0	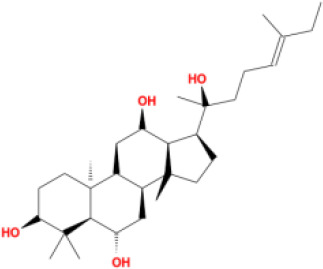
M30	ginsenoside Rg3_qt	*Panax ginseng* C.A.Mey	29.70	0.34	0.77	0	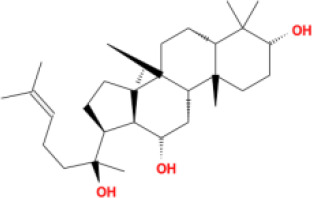
M31	ginsenosideRh4_qt	*Panax ginseng* C.A.Mey	9.84	0.36	0.78	0	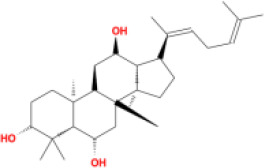
M32	ginsenoside Rs1_qt	*Panax ginseng* C.A.Mey	11.87	-0.86	0.46	0	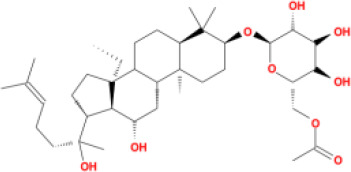
M33	hypaconitine	*Aconitum carmichaeli* Debeaux	31.39	-0.34	0.26	19.87	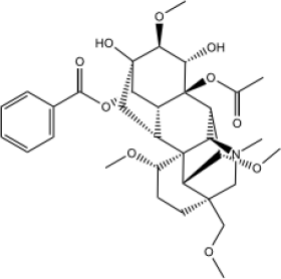
M34	Benzoylmesaconine	*Aconitum carmichaeli* Debeaux	8.55	-0.52	0.27	0	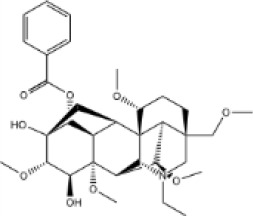
M35	karakoline	*Aconitum carmichaeli* Debeaux	51.73	0.32	0.73	11.10	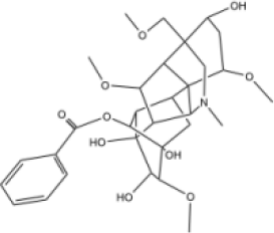
M36	neojiangyouaconitine	*Aconitum carmichaeli* Debeaux	9.83	0.01	0.26	0	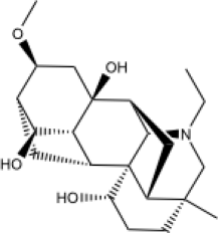
M37	benzoylhypaconine	*Aconitum carmichaeli* Debeaux	8.70	-0.29	0.29	0	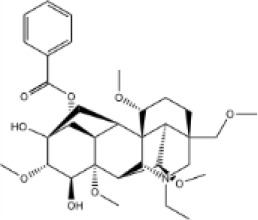
M38	benzoylnapelline	*Aconitum carmichaeli* Debeaux	34.05	0.19	0.52	15.7	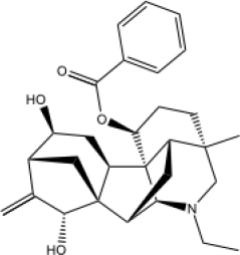
M39	6-demethyldesoline	*Aconitum carmichaeli* Debeaux	51.87	-0.26	65	13.1	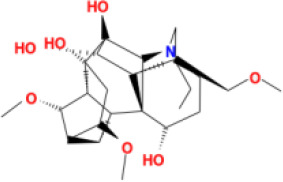
M40	deoxyaconitine	*Aconitum carmichaeli* Debeaux	30.95	-0.23	0.24	22.6	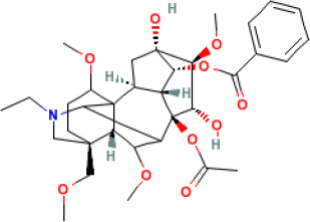
M41	ignavine	*Aconitum carmichaeli* Debeaux	84.07	-0.07	0.24	28.9	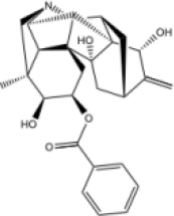
M42	isotalatizidine	*Aconitum carmichaeli* Debeaux	50.82	-0.11	0.73	11.5	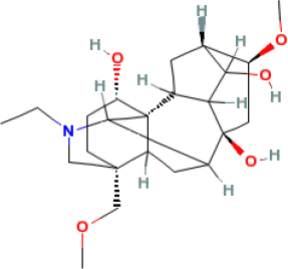
M43	aconitine	*Aconitum carmichaeli* Debeaux	7.87	-0.58	0.23	0	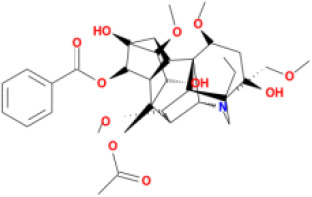
M44	mutatochrome	*Rheum palmatum* L.	48.64	1.97	0.61	15.7	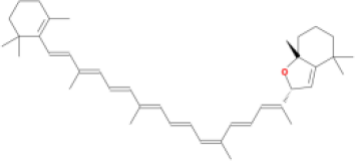
M45	rheinoside A	*Rheum palmatum* L.	0.82	-3.17	0.68	0	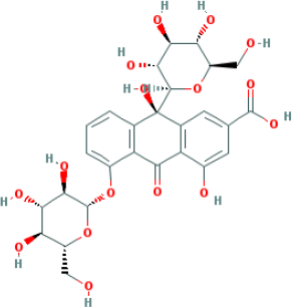
M46	sennoside C	*Rheum palmatum* L.	3.99	-3.53	0.08	0	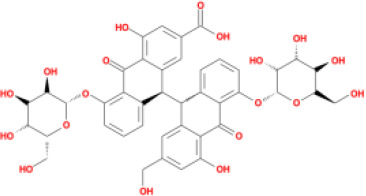
M47	rheosmin	*Rheum palmatum* L.	26.79	0.97	0.04	0	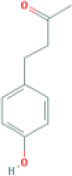
M48	aloeemodin	*Rheum palmatum* L.	20.65	-0.22	0.24	0	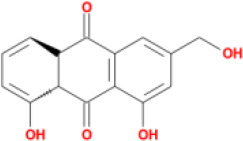
M49	palmidin A	*Rheum palmatum* L.	32.45	-0.36	0.65	32.1	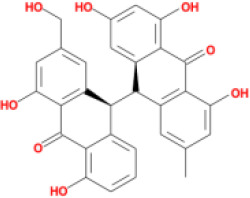

### Target Prediction of Potential Compounds

In order to reveal the interactions between all candidate compounds and the target proteins on a large scale, the WES model was employed to calculate and forecast the targets. Based on the results of simulation, 64 potential targets were identified for the 49 candidates (**Supplementary Table S2**). The full names of these symbols of targets were listed in **Supplementary Table S3**.

### Network Analysis

#### Compound-Target Network

**Figure 1:**
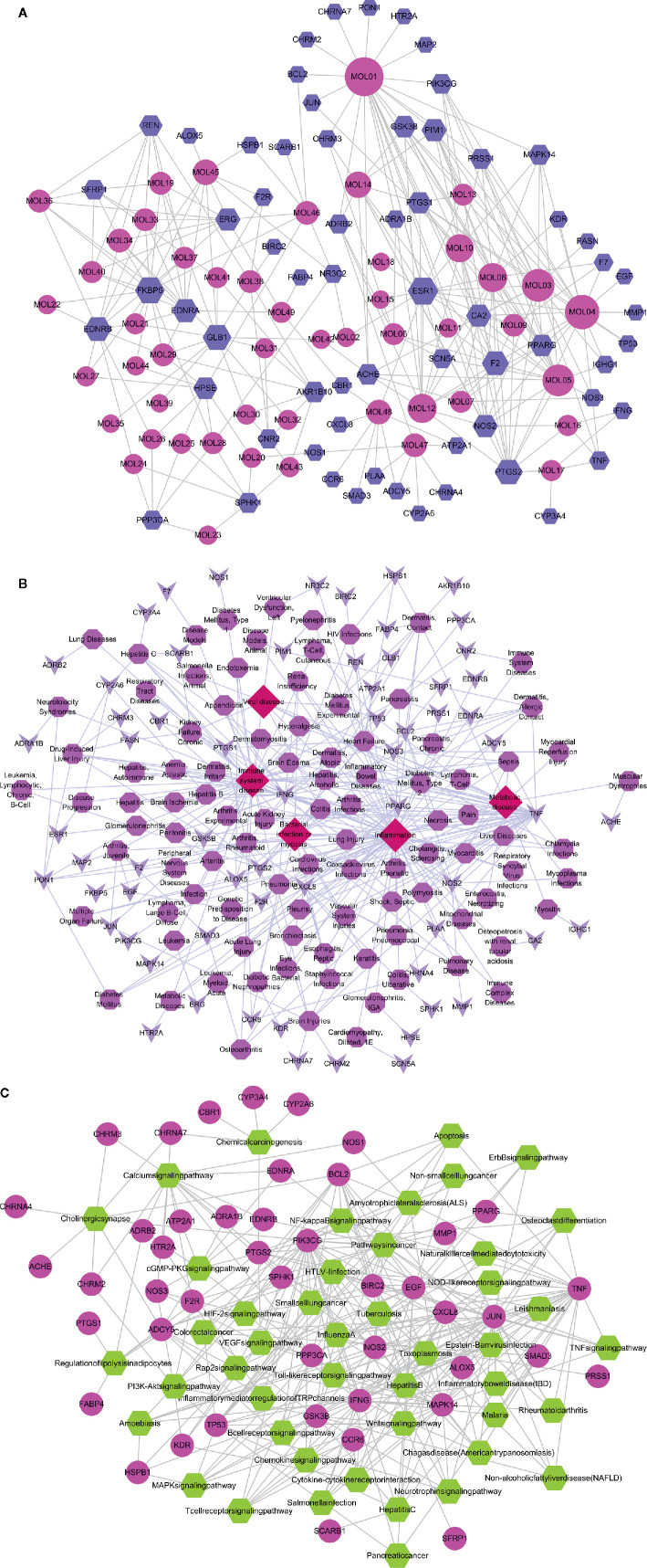
Compound-target-disease-pathway networks. **(A)** Compound-target network of SFH consisting of 113 nodes and 291 interactions. **(B)** Target-disease network including 46 candidate targets and 5 important diseases. **(C)** Target-pathway network including 45 candidate targets and 46 KEGG pathways.

The compounds of SFH acted on multiple targets, and each target was involved in various physiological and pathological processes. The compound-target network includes 113 nodes and 291 interactions. A total of 64 target proteins were screened out, which may be related to inflammatory response, coagulation disorder, and tissue damages. Compounds such as sitosterol, emodin, chrysophanol, and deltoin had the highest weight and number of targets. Additionally, retrieval of multiple relationships between the compounds and targets showed that proteins including GSK3β, ESR1, PPARG, PTGS2, AKR1B10, and MAPK14 may act as potential targets of SFH and impact downstream signaling pathways (**Figure 1A**).

#### Target-Diseases Network

To understand the role of targets of SFH on diseases, we established a target-disease network using PharmGKB, Drugbank, and TTD databases. Total of 46 targets were directly involved in immune system disease, while nine targets were related to inflammation. Moreover, viral disease, bacterial infection/mycosis, and metabolic disease were also highly linked to the potential targets (**Figure 1B**).

#### Target-Pathway Network

To further investigate the intervention of SFH on various diseases, KEGG and DAVID databases were used to identify the relationship between the potential targets and related signaling pathways. The target-pathway interaction network comprised of 45 targets and 46 pathways, which included 91 nodes and 293 edges. Most of the predicted targets were involved in pathways that contributed to diseases. These high-weight signaling pathways included Toll-like receptor, MAPK, JAK/STAT, PPAR, VEGF, NOD-like receptor and NF-kappa B signaling pathways, which are closely related to sepsis, infection immunity, inflammatory response, coagulation function, organ damage, immune disorders and other diseases (**Figure 1C**).

Details of the topological properties of the networks in **Supplementary Table S4**.

#### Integration of Networks

**Figure 2:**
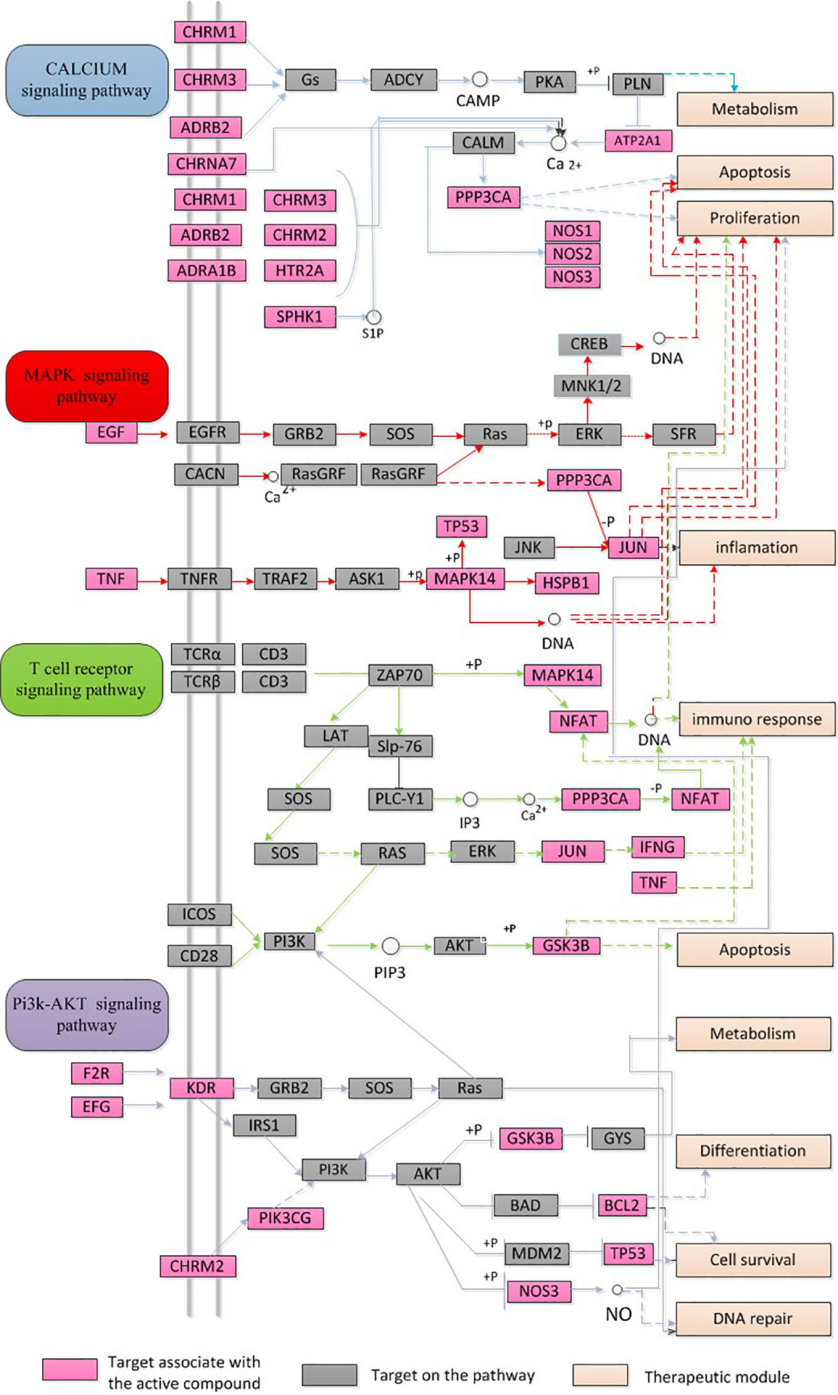
Integration of networks of SFH targets. Sepsis-related pathway including calcium signaling pathway, MAPK signaling pathway, T cell receptor signaling pathway, and PI3K-AKT signaling pathway.

To systematically estimate the synergistic effects of these three herbal ingredients of SFH on sepsis, an integrated “sepsis-related pathway” approach was structured based on the current data on sepsis. The targets of SFH were associated with key pathological processes of sepsis, including the calcium signaling pathway, MAPK signaling pathway, T cell receptor signaling pathway, and PI3K-AKT signaling pathway (**Figure 2**).

### Identification of Major Components of SFH Formula

**Figure 3:**
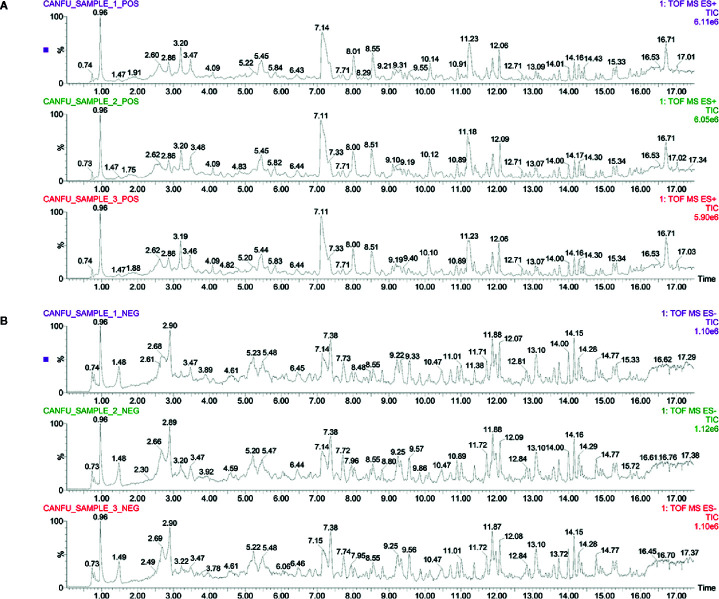
Identification of major components of SFH formula. Three parallel samples of SFH formula were detected by employing the UPLC–MS/MS system. Data were collected and proceeded by software Masslynx 4.1. The positive **(A)** and negative **(B)** ion chromatograms of SFH formula were shown as indicated.

This study employed UPLC–MS/MS detection to investigate the ingredients and evaluate the repeatability and stability of SFH formula. As shown in **Figure 3**, the total ion chromatogram of three parallel samples illustrated the composition and percentage of ingredients in the SFH formula. Moreover, data based on the characteristic peaks found that the SFH formula showed high repeatability and stability according to coefficient correlation. The contents of major compounds were further examined by using UPLC–MS/MS. Data showed that main compounds including aloe-emodin (48.13 μg/ml), rhein (110.69 μg/ml), fuziline (68.78 μg/ml), deoxyaconitine (12.08 μg/ml), ginsenoside rh2 (1.74 μg/ml), quercetin (2.01 μg/ml), gallic acid (157.47 μg/ml) were detected in SFH formula, details in **Supplementary Table S5**.

### Effect of SFH on Zebrafish Model

#### Effect of SFH on Poly (I:C)-Induced Infection

**Figure 4:**
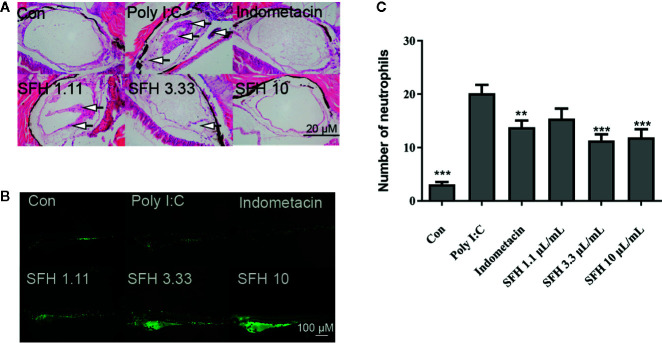
Effect of SFH on Poly (I:C)-induced pneumonia. **(A)** HE staining to observe the pathological features. Arrows mark the inflammatory infiltration and cell shedding. **(B)** Fluorescence detection on neutrophils. **(C)** The numbers of neutrophils in air bladder tissue are counted. For all experiments, at least 30 larvae were used for each condition. ** indicated significant difference at p < 0.01, *** indicated significant difference at p < 0.001, compared with Poly (I:C) group.

Neutrophils are important immunocytes in the innate immunity system. We investigated the effect of SFH on neutrophil activation using a Poly (I:C)-induced zebrafish infection model. Obvious inflammatory infiltration and cell shedding were observed in the air bladder of Poly (I:C) stimulated group. However, SFH treatment at doses of 3.33 and 10 mg/ml notably improved the symptoms, with no difference with indomethacin group (**Figure 4A**). Moreover, data showed that the number of neutrophils were markedly increased when challenged by Poly (I:C), compared with the control group. Treatment with indomethacin as a positive control notably suppressed the generation of neutrophils induced by Poly (I:C) (p < 0.05). SFH treatment at doses of 3.33 and 10 mg/ml could significantly inhibit the production of neutrophils, compared with the Poly (I:C) group (p < 0.001). Moreover, SFH at these two doses exhibited a better treatment effect than indomethacin (p < 0.05) (**Figures 4B, C**).

### Effect of SFH on Macrophage Activation

**Figure 5:**
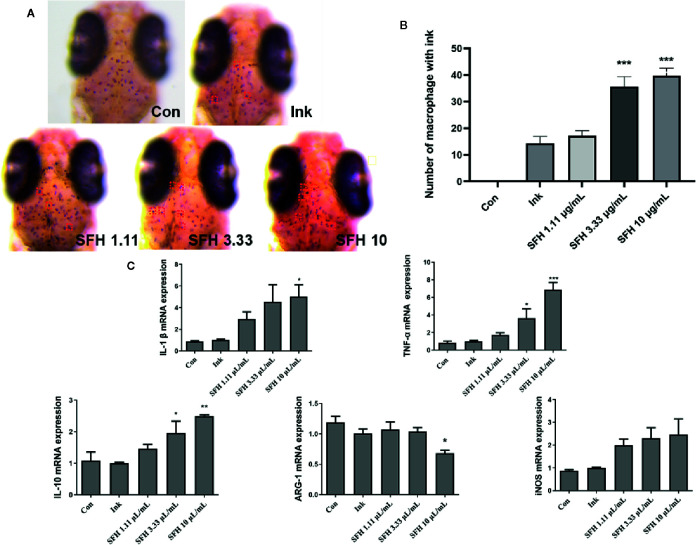
Effect of SFH on macrophage activation. **(A)** Observation of macrophages with or without ink under dissecting microscope. **(B)** The numbers of macrophages with ink are counted. For all experiments, at least 30 larvae were used for each condition. *** indicated significant difference at p < 0.001, compared with ink group. **(C)** RT-PCR detection on the mRNA expression of M1/M2 markers in macrophage. For all experiments, at least 30 larvae were used for each condition. * indicated significant difference at p < 0.05, ** indicated significant difference at p < 0.01, *** indicated significant difference at p < 0.001, compared with ink group.

Macrophages play a key role in sepsis by phagocytosis of pathogens and inflammatory response. We used ink to establish a macrophage phagocytosis model. Data showed that SFH treatment significantly enhanced the phagocytic capacity of macrophages by increasing the number of macrophages that engulfed ink (**Figures 5A, B**). The expression of several key phenotypic indexes of macrophages represents their functional activation. Hence, we detected the mRNA expression levels of polarization biomarkers such as TNF-α, iNOS, IL-1β, IL-10, and Arg-1 in macrophages of zebrafish treated by SFH. SFH treatment significantly enhanced the mRNA expression of TNF-α at doses of 3.33 mg/ml (p < 0.05) and 10 mg/ml (p < 0.001), compared with the model group. The expression levels of IL-1β and IL-10 were also increased after treatment with SFH at the dose of 10 mg/ml (p < 0.05). However, the expression of AGR-1 was suppressed by SFH treatment at the dose of 10 mg/ml (p < 0.05). SFH had limited regulatory effect on iNOS expression (**Figure 5C**).

### Effect of SFH on Coagulation Function

**Figure 6:**
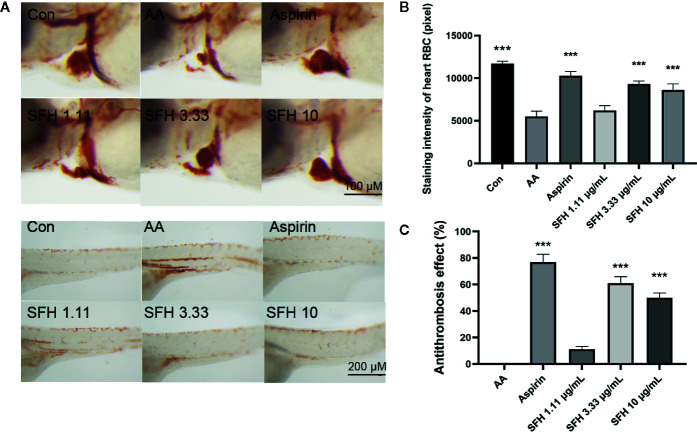
Effect of SFH on coagulation function. **(A)** Observation of RBC in the heart and trunk of zebrafish under dissecting microscope. **(B)** Detection of staining intensity of RBC in the heart of zebrafish using dissecting microscope. For all experiments, at least 30 larvae were used for each condition. *** indicated significant difference at p < 0.001, compared with AA group. **(C)** Evaluation of the antithrombosis effect of SFH. For all experiments, at least 30 larvae were used for each condition. *** indicated significant difference at p < 0.001, compared with AA group.

To investigate the role of SFH in coagulation function, we evaluated the staining intensity of red blood cells (RBC) in the heart and trunk of zebrafish. Compared with the control group, treatment with arachidonic acid (AA) significantly decreased the number of red blood cells in the heart but increased their number in the trunk (p < 0.001). However, SFH treatment at doses of 3.33 and 10 mg/ml effectively enhanced the generation of red blood cells in the heart (p < 0.001). Treatment with aspirin as a positive control showed a similar effect to SFH (p > 0.05) (**Figures 6A, B**). By using the model of zebrafish thrombosis assay technology, we found that SFH treatment at doses of 3.33 and 10 mg/ml had promising antithrombosis properties, compared with the aspirin treatment (p > 0.05) (**Figure 6C**).

## Discussion

The outbreak of COVID-19 caused by SARS-CoV-2 has resulted in an acute respiratory illness pandemic worldwide. A global health emergency was announced by the WHO Emergency Committee on January 30, 2020, based on the growing number of infected and dead patients in China and several other countries. The lack of knowledge of this virus in the initial stage resulted in large numbers of infected patients, with approximately 5% mortality rate. Extensive efforts of clinicians and scientists worldwide have gradually uncovered several critical aspects of this disease. It has been well-documented that critically ill COVID-19 patients accord with the Sepsis-3.0 guideline. Moreover, negative results for bacteria and fungus based on specimen cultures were observed in the lower respiratory tract and blood samples of 76% of sepsis patients, indicating that severe patients are more likely to suffer from viral sepsis (Lin et al., 2018; Zhou et al., 2020).

Sepsis is one of the complications in critical patients. The pathophysiological process of sepsis is complex. Excessive activation of inflammation in the early stage of sepsis contributes to unexpected immune response and tissue damages. However, with the increase of inflammatory mediators and anti-inflammatory reaction *in vivo*, the host gradually reaches a state of immunosuppression, which leads to immune function disorder, apoptosis, necrosis and coagulation dysfunction. The progression of COVID-19 infection is also characterized by these features. Despite the pulmonary injury, the production of pro-inflammatory cytokines including IL-1β, IL-6, TNF-α, macrophage inflammatory protein 1-α, interferon gamma-induced protein-10, and monocyte chemoattractant protein-1 were significantly enhanced in COVID-19 patients (Huang et al., 2020; Liu et al., 2020). Besides, 71.4% of dead patients matched the grade of disseminated intravascular coagulation (DIC) according to the International Society on Thrombosis and Hemostasis criteria, and showed abnormal coagulation in later stages of the disease (Tang et al., 2020; Zhou et al., 2020).

SFH formula, which is composed of *Panax ginseng C.A.Mey*, *Aconitum carmichaeli Debeaux*, and *Rheum palmatum L.*, was designed for treating septic syndrome and used for treating COVID-19 patients. In addition to the well-published LianHuaQingWen capsule (Hu et al., 2020), many unreported TCMs are effective for COVID-19 and its complications, including SFH formula. Chinese herbs have complex mechanism and pharmacology, with multi-component, multi-target, and multi-channel systems. Systems pharmacology has been used to study TCMs by screening the active compounds, predicting the targets, and analyzing the potential compound-target-disease relationships. In this study, we screened 49 active candidates from SFH formula using indicators such as OB, DL, Caco-2, and HL. There are some typical pharmacodynamic molecules who are not up to standard, possessing well-documented biological activities established in *in-vivo* and *in-vitro* studies. For example, emodin (M4) from *Rheum palmatum L.* has been reported to exhibit promising anti-inflammatory, anti-oxidative, hepatoprotective, and anticancer effects (Dong et al., 2016). Again as an example, chrysophanol (M5) from *Rheum palmatum L.* has been established to have anticancer, antioxidation, neuroprotection, antibacterial and antiviral properties (Xie et al., 2019). Therefore, by retrieving in DrugBank and NCBI database, we listed these compounds as typical pharmacodynamic candidates of SFH formula.

In-depth analysis revealed many compounds with well-established pharmacological effects. For example, emodin from *Rheum palmatum L.* has been proven to possess hepatoprotective, anti-inflammatory, antioxidant, anticoagulant, and anti-microbial activities (Nemmar et al., 2015; Dong et al., 2016). Ginsenoside rh2 from *Panax ginseng C.A.Mey* exhibits antimicrobial resistance and cure for pneumonia (Hsieh et al., 2018; Liu et al., 2018). Benzoylmesaconine from *Aconitum carmichaeli Debeaux* has antiviral and anti-nociceptive activities (Suzuki et al., 1994; Kobayashi et al., 2003). To further investigate the role of these 49 candidates, we predicted their potential targets using the WES model. A total of 64 targets were identified, including GSK3β, ESR1, PPARG, PTGS2, AKR1B10, and MAPK14. Evaluation of the roles of these targets suggested that they may be involved in the therapeutic effect of SFH on sepsis. PPARG has been proven to regulate macrophage activity in inflammatory response (Majithia et al., 2016). GSK3β is active in many important signaling pathways, including cell proliferation, migration, inflammation, immune response, and apoptosis (Patel and Woodgett, 2017). The MAPK superfamily members, such as MAPK14, are key regulators of macrophage inflammation, autophagy and cell proliferation (He et al., 2018; Wu et al., 2019). The target-disease association network further illustrated that the targets of SFH may be involved in inflammatory disease, viral disease, bacterial infection/mycosis, and metabolic disease. These pathological processes have been reported to have a strong relationship with sepsis (Van der Poll et al., 2017; Cecconi et al., 2018; Lin et al., 2018; Van Wyngene et al., 2018). To systematically understand how SFH formula exhibits curative effect on sepsis, we summarized an integrated “sepsis-related pathway” approach. The pathways regulated by SFH, including calcium signaling pathway, MAPK signaling pathway, T cell receptor signaling pathway, and PI3K-AKT signaling pathway, contribute to the development of sepsis.

Current studies reported that severe COVID-19 patients with septic syndrome mainly showed abnormal pathological features, including virus infection and tissue damage, excessive inflammation in early stage but immune suppression in late stage, and coagulation dysfunction (Li et al., 2020). Since the data of systems pharmacology illustrated that SFH may regulate several key targets and biological processes of sepsis, such as PPARG in inflammatory response, GSK3β and MAPK14 in cell proliferation, and PTGS2 in coagulation, we hypothesized that SFH improves the condition of critically ill COVID-19 patients with septic syndrome by ameliorating lung injury, suppressing excessive inflammation but enhancing the capacity of pathogen phagocytosis and killing, and improving the function of blood coagulation. Therefore, we employed various zebrafish models to test our hypothesis. We first simulated SARS-CoV-2 pulmonary infection using polyinosinic-polycytidylic acid (poly I:C), a synthetic double-stranded RNA immune-stimulant for study of SARS-CoV-2 infection (Kumaki et al., 2017; Gao et al., 2020). It is documented that hyper-inflammation due to neutrophils occurs in viral infections of the upper respiratory tract (Drescher and Bai, 2013). Recent studies also reported that COVID-19 lung injury in some patients might involve dysregulated neutrophil activity (Didangelos, 2020). Thus, inhibition of excessive neutrophil activation may help to control the lung damage. Our data showed that Poly I:C stimulation significantly increased the number of neutrophils in the air bladder of zebrafish. However, SFH treatment ameliorated the hyper-inflammation in air bladder tissue by suppressing the neutrophil infiltration. Macrophages, as innate immune cells, are the principal players in viral infections (Jakubzick et al., 2017). Several observational studies have characterized over-activation of monocytes in the early stage of SARS-CoV-2 infection (Pence, 2020). However, studies involving severe and critically ill COVID-19 patients demonstrated a substantial decrease in circulating monocytes and a sudden decrease in their expression of antigen (HLA)-DR (Giamarellos-Bourboulis et al., 2020; Sanchez-Cerrillo et al., 2020). These data further support the macrophage dysfunction and adaptive immune system impairments, which may lead to worsened medical condition or death. According to our data, SFH treatment promoted M1 macrophage activation by enhancing the expression of TNF-α, iNOS, IL-1β, and IL-10, while suppressing ARG-1 expression. This finding offers additional methods for treating viral infection patients with immune dysfunction. Coagulation dysfunction is another important pathogenesis of COVID-19 patients with septic syndrome. Coagulation function in SARS-CoV-2 infected patients is notably impaired compared with normal patients (Guevara-Noriega et al., 2020). Disseminated intravascular coagulation (DIC) with excessive thrombin generation poses a challenge to clinical therapeutics of COVID-19. We used a zebrafish thrombus model to evaluate the anti-thrombosis property of SFH. Results showed that SFH treatment significantly suppressed AA-induced generation of thrombus. Thus, SFH treatment may help to improve the coagulopathy of COVID-19 patients.

COVID-19 is an emerging and rapidly evolving pandemic. Traditional Chinese herbs have played important roles in treating SARS-CoV-2 infection in China. SFH treatment showed promising therapeutic effects in critically ill COVID-19 patients. This study delineated the molecular mechanism of SFH using systems pharmacology tools and *in-vivo* zebrafish models. The results provide additional evidence of TCMs as complementary and alternative therapies for treating COVID-19.

## Data Availability Statement

The raw data supporting the conclusions of this article will be made available by the authors, without undue reservation, to any qualified researcher.

## Ethics Statement

The animal study was reviewed and approved by Committee for the Care and Use of Experimental Animals at Beijing Institute of Traditional Chinese Medicine.

## Author Contributions

TL: substantial contributions to the conception and design of the work, drafting the work, and revising it critically for important intellectual content. YG: substantial contributions to the design of the work, interpretation of data for the work. SH, JZ, YB, and NW: acquisition, analysis, and interpretation of data for the work. YL: drafting the work and revising it critically for important intellectual content. QL: language polishing and adjustment of structure of manuscript. XX: substantial contributions to the design of the work, final approval of the version to be published, agreement to be accountable for all aspects of the work in ensuring that questions related to the accuracy and integrity of any part of the work are appropriately investigated and resolved.

## Funding

This work was supported by grants from the National Natural Science Foundation of China (81673934, 62041701, 81973608), the National Major Scientific and Technological Project (2017ZX10305501), the Beijing Natural Science Foundation (No. 7192083).

## Conflict of Interest

The authors declare that the research was conducted in the absence of any commercial or financial relationships that could be construed as a potential conflict of interest.
