# Supplementary material for: Systems Pharmacology and Verification of ShenFuHuang Formula in Zebrafish Model Reveal Multi-Scale Treatment Strategy for Septic Syndrome in COVID-19
Source: Front Pharmacol. 2020 Sep 15;11:584057. doi: 10.3389/fphar.2020.584057 (PMC7523021; doi:10.3389/fphar.2020.584057)
Supplement: Supplementary file 1 [file Table_1.docx]

**Supplementary Table S1**

Primer Sequences

| **Gene** | **5’→3’** | |
| --- | --- | --- |
| ***β-actin*** | Forward | TCGAGCAGGAGATGGGAACC |
|  | Reverse | CTCGTGGATACCGCAAGATTC |
| ***TNF-α*** | Forward | ATCTTCAAAGTCGGGTGTATG |
|  | Reverse | TGTGCCCAGTCTGTCTCC |
| ***iNOS*** | Forward | CCTCCTCATGTACCTGAATCTCG |
|  | Reverse | GCTCCTTGCTTTAGTATGTCGC |
| ***ARG-1*** | Forward | TGGGAATAATAGGCGCTCCGTTC |
|  | Reverse | TCCTTCACCACACAACCTTGC |
| ***IL-10*** | Forward | TTCAGGAACTCAAGCGGGAT |
|  | Reverse | AAGAGCAAATCAAGCTCCCCC |
| ***IL-1β*** | Forward | GTCACACTGAGAGCCGGAAG |
|  | Reverse | GCAGGCCAGGTACAGGTTAC |
