# Supplementary material for: Systems Pharmacology and Verification of ShenFuHuang Formula in Zebrafish Model Reveal Multi-Scale Treatment Strategy for Septic Syndrome in COVID-19
Source: Front Pharmacol. 2020 Sep 15;11:584057. doi: 10.3389/fphar.2020.584057 (PMC7523021; doi:10.3389/fphar.2020.584057)
Supplement: Supplementary file 2 [file Table_2.docx]

**Supplementary Tables S2**

**Targets of candidates**

| Compound | Target | |
| --- | --- | --- |
|  | Human | Mouse |
| M1 | BCL2、PON1、JUN、MAP2、NOS2、PTGS1、CHRM3、F2、ESR1、SCN5A  PPARG、PTGS2、CA2、ACHE、HTR2A  CHRM2、ADRA1B、ADRB2、MAPK14  GSK3B、PIK3CG、CHRNA7、PRSS1  PIM1 | BCL2、PON1、JUN、MAP2、NOS2、PTGS1、CHRM3、F2、ESR1、SCN5A、PPARG、PTGS2、CA2 |
| M2 | ESR1、NR3C2 | ESR1、NR3C2 |
| M3 | TNF、FASN、TP53、NOS2、TGS1、ESR1  PPARG、PTGS2、NOS3、CA2、ACHE  MAPK14、GSK3B、PIK3CG、IGHG1  PRSS1、PIM1 | TNF、FASN、TP53、NOS2、TGS1、ESR1  PPARG、PTGS2、NOS3、CA2、ACHE、MAPK14、GSK3B、PIK3CG、PIM1 |
| M4 | NOS2、KDR、TNF、MMP1、TP53、EGF、PTGS1、F2、ESR1、PPARG  PTGS2、CA2、F7、MAPK14、GSK3B  PIK3CG、IGHG1、PRSS1、PIM1 | NOS2、KDR、TNF、MMP1、TP53、EGF、PTGS1、F2、ESR1、CA2、F7、MAPK14、GSK3B  PIK3CG、PRSS1、PIM1 |
| M5 | NOS2、PTGS1、F2、ESR1、SCN5A  PPARG、PTGS2、NOS3、CA2、ACHE  MAPK14、GSK3B、PIK3CG、IGHG1、PRSS1、PIM1 | NOS2、PTGS1、F2ESR1、SCN5A、PPARG、PTGS2、NOS3、CA2、PRSS1、PIM1 |
| M6 | CHRM3、F2、SCN5A、ADRA1B  ADRB2 | CHRM3、F2、SCN5A、ADRA1B、ADRB2 |
| M7 | F2、PPARG、NOS3、CA2、ACHE | F2、PPARG、NOS3、CA2、ACHE |
| M8 | NOS2、F2、ESR1、SCN5A、PPARG  PTGS2、CA2、F7、KDR、MAPK14  GSK3B、PRSS1、PIM1 | NOS2、F2、ESR1  SCN5A、PPARG  PTGS2、CA2、F7、KDR、MAPK14、GSK3B、PRSS1、PIM1 |
| M9 | F2、ESR1、PTGS2、CA2、F7、PIM1 | F2、ESR1、PTGS2、CA2、F7、PIM1 |
| M10 | JUN、NOS2、PTGS1、F2、ESR1  PPARG、PTGS2、CA2、MAPK14  GSK3B、PIK3CG、PIM1 | JUN、NOS2、PTGS1  ESR1、PPARG、PTGS2、CA2、MAPK14、GSK3B、PIK3CG、PIM1 |
| M11 | F2、ESR1 | F2、ESR1 |
| M12 | NOS2、PTGS1、F2、ESR1、SCN5A、PPARG、PTGS2、CA2、ACHE、ADRA1B、ADRB2、GSK3B、PRSS1 | NOS2、PTGS1、F2、ESR1、SCN5A、PPARG、PTGS2、CA2、ACHE、ADRA1B、ADRB2、GSK3B、PRSS1 |
| M13 | PTGS1、ESR1、PTGS2、PIK3CG  PIM1 | PTGS1、ESR1、PTGS2、PIK3CG、PIM1 |
| M14 | NOS2、PTGS1、CHRM3、F2、ESR1、PTGS2、ACHE、ADRB2、GSK3B  PIM1 | NOS2、PTGS1、CHRM3、F2、ESR1、PTGS2、ACHE、ADRB2、GSK3B  PIM1 |
| M15 | ESR1 | ESR1 |
| M16 | NOS2、PTGS2、TNF、IFNG | NOS2、PTGS2、TNF、IFNG |
| M17 | PTGS2、TNF、IFNG、CYP3A4 | PTGS2、TNF、IFNG、CYP3A4 |
| M18 | ESR1 | ESR1 |
| M19 | EDNRA、REN、SFRP1、ERG、GLB1  FKBP5 | EDNRA、SFRP1 |
| M20 | SPHK1、NOS1、HPSE | NOS1、HPSE |
| M21 | EDNRA、GLB1、EDNRB、FKBP5 | EDNRA、EDNRB |
| M22 | EDNRB、FKBP5 | EDNRB |
| M23 | SPHK1、PPP3CA | SPHK1、PPP3CA |
| M24 | SPHK1、EDNRB、PPP3CA | SPHK1、EDNRB、PPP3CA |
| M25 | SPHK1、HPSE、PPP3CA | SPHK1、HPSE、PPP3CA |
| M26 | HPSE、EDNRB、PPP3CA | HPSE、EDNRB、PPP3CA |
| M27 | HPSE、EDNRB、FKBP5 | HPSE、EDNRB |
| M28 | SPHK1、NR3C2、HPSE、PPP3CA  FKBP5 | SPHK1、NR3C2、HPSE、PPP3CA |
| M29 | AKR1B10、EDNRA、HPSE、GLB1、EDNRB、FKBP5 | EDNRA、HPSE、EDNRB |
| M30 | AKR1B10、CNR2、GLB1 | CNR2 |
| M31 | AKR1B10、CNR2、GLB1 | CNR2 |
| M32 | AKR1B10、CNR2、GLB1 | CNR2 |
| M33 | EDNRA、REN、SFRP1、ERG、EDNRB  FKBP5 | EDNRA、EDNRB |
| M34 | EDNRA、REN、SFRP1、ERG、GLB1  EDNRB、FKBP5 | EDNRA、SFRP1、EDNRB |
| M35 | GLB1 | GLB1 |
| M36 | EDNRA/SFRP1/ERG、REN、EDNRB  FKBP5 | EDNRB、EDNRA/SFRP1 |
| M37 | EDNRA、ERG、REN、EDNRB  FKBP5 | EDNRA、EDNRB |
| M38 | AKR1B10、CNR2、F2R、BIRC2、REN  GLB1 | F2R、BIRC2 |
| M39 | GLB1 | GLB1 |
| M40 | EDNRA、REN、SFRP1、ERG、FKBP5 | EDNRA、SFRP1、ERG |
| M41 | AKR1B10、SFRP1、F2R、GLB1 | SFRP1、F2R、GLB1 |
| M42 | GLB1、ACHE | GLB1、ACHE |
| M43 | AKR1B10、SPHK1、CNR2 | SPHK1 |
| M44 | HPSE、FKBP5 | HPSE |
| M45 | EDNRA、NR3C2、F2R、ERG、GLB1  ALOX5、EDNRB、HSPB1、FKBP5 | EDNRA、NR3C2、F2R、ERG、GLB1、ALOX5、EDNRB、HSPB1、FKBP5 |
| M46 | NR3C2、SCARB1、ERG、HPSE、HSPB1  FKBP5、BCL2 | NR3C2、HPSE、HSPB1 |
| M47 | ATP2A1、NOS1、ADRB2、CYP2A6  ADCY5、PLAA、CHRNA4 | ATP2A1、NOS1、ADRB2、CYP2A6、CHRNA4 |
| M48 | ATP2A1、CCR6、ADCY5、CBR1  SMAD3、ESR1、PLAA、CXCL8 | ATP2A1、ESR1 |
| M49 | FABP4、EDNRA、ERG、CBR1 | EDNRA、ERG、CBR1 |
