# Supplementary material for: Systems Pharmacology and Verification of ShenFuHuang Formula in Zebrafish Model Reveal Multi-Scale Treatment Strategy for Septic Syndrome in COVID-19
Source: Front Pharmacol. 2020 Sep 15;11:584057. doi: 10.3389/fphar.2020.584057 (PMC7523021; doi:10.3389/fphar.2020.584057)
Supplement: Supplementary file 3 [file Table_3.docx]

**Supplementary Tables S3**

**Full name of symbol**

| **Gene Symbol** | **Entrez ID** | **Gene Name** |  |  |  |  |
| --- | --- | --- | --- | --- | --- | --- |
| CYP3A4 | 1576 | cytochrome P450 family 3 subfamily A member 4(CYP3A4) | | | | |
| IGHG1 | 3500 | immunoglobulin heavy constant gamma 1 (G1m marker)(IGHG1) | | | | |
| PTGS2 | 5743 | prostaglandin-endoperoxide synthase 2(PTGS2) | | | | |
| ADCY5 | 111 | adenylate cyclase 5(ADCY5) | | |  |  |
| PPARG | 5468 | peroxisome proliferator activated receptor gamma(PPARG) | | | | |
| PTGS1 | 5742 | prostaglandin-endoperoxide synthase 1(PTGS1) | | | | |
| MMP1 | 4312 | matrix metallopeptidase 1(MMP1) | | | |  |
| GLB1 | 2720 | galactosidase beta 1(GLB1) | | |  |  |
| EDNRA | 1909 | endothelin receptor type A(EDNRA) | | | |  |
| EDNRB | 1910 | endothelin receptor type B(EDNRB) | | | |  |
| HPSE | 10855 | heparanase(HPSE) | |  |  |  |
| IFNG | 3458 | interferon gamma(IFNG) | | |  |  |
| CHRNA4 | 1137 | cholinergic receptor nicotinic alpha 4 subunit(CHRNA4) | | | | |
| NOS3 | 4846 | nitric oxide synthase 3(NOS3) | | |  |  |
| CHRNA7 | 1139 | cholinergic receptor nicotinic alpha 7 subunit(CHRNA7) | | | | |
| NOS2 | 4843 | nitric oxide synthase 2(NOS2) | | |  |  |
| PIK3CG | 5294 | phosphatidylinositol-4,5-bisphosphate 3-kinase catalytic subunit  gamma(PIK3CG) | | | | |
| ESR1 | 2099 | estrogen receptor 1(ESR1) | | |  |  |
| TP53 | 7157 | tumor protein p53(TP53) | | |  |  |
| PIM1 | 5292 | Pim-1 proto-oncogene, serine/threonine kinase(PIM1) | | | | |
| F7 | 2155 | coagulation factor VII(F7) | | |  |  |
| ADRB2 | 154 | adrenoceptor beta 2(ADRB2) | | |  |  |
| CCR6 | 1235 | C-C motif chemokine receptor 6(CCR6) | | | |  |
| CHRM3 | 1131 | cholinergic receptor muscarinic 3(CHRM3) | | | |  |
| CHRM2 | 1129 | cholinergic receptor muscarinic 2(CHRM2) | | | |  |
| AKR1B10 | 57016 | aldo-keto reductase family 1 member B10(AKR1B10) | | | | |
| JUN | 3725 | Jun proto-oncogene, AP-1 transcription factor subunit(JUN) | | | | |
| F2 | 2147 | coagulation factor II, thrombin(F2) | | | |  |
| CYP2A6 | 1548 | cytochrome P450 family 2 subfamily A member 6(CYP2A6) | | | | |
| HSPB1 | 3315 | heat shock protein family B (small) member 1(HSPB1) | | | | |
| CA2 | 760 | carbonic anhydrase 2(CA2) | | |  |  |
| ACHE | 43 | acetylcholinesterase (Cartwright blood group)(ACHE) | | | | |
| TNF | 7124 | tumor necrosis factor(TNF) | | |  |  |
| FKBP5 | 2289 | FK506 binding protein 5(FKBP5) | | |  |  |
| PRSS1 | 5644 | protease, serine 1(PRSS1) | | |  |  |
| NR3C2 | 4306 | nuclear receptor subfamily 3 group C member 2(NR3C2) | | | | |
| CXCL8 | 3576 | C-X-C motif chemokine ligand 8(CXCL8) | | | |  |
| PLAA | 9373 | phospholipase A2 activating protein(PLAA) | | | |  |
| CBR1 | 873 | carbonyl reductase 1(CBR1) | | |  |  |
| BCL2 | 596 | BCL2, apoptosis regulator(BCL2) | | |  |  |
| REN | 5972 | renin(REN) | |  |  |  |
| CNR2 | 1269 | cannabinoid receptor 2(CNR2) | | |  |  |
| FASN | 2194 | fatty acid synthase(FASN) | | |  |  |
| SCARB1 | 949 | scavenger receptor class B member 1(SCARB1) | | | | |
| PPP3CA | 5530 | protein phosphatase 3 catalytic subunit alpha(PPP3CA) | | | | |
| EGF | 1950 | epidermal growth factor(EGF) | | |  |  |
| SCN5A | 6331 | sodium voltage-gated channel alpha subunit 5(SCN5A) | | | | |
| ERG | 2078 | ERG, ETS transcription factor(ERG) | | | |  |
| NOS1 | 4842 | nitric oxide synthase 1(NOS1) | | |  |  |
| SPHK1 | 8877 | sphingosine kinase 1(SPHK1) | | |  |  |
| SMAD3 | 4088 | SMAD family member 3(SMAD3) | | | |  |
| BIRC2 | 329 | baculoviral IAP repeat containing 2(BIRC2) | | | |  |
| KDR | 3791 | kinase insert domain receptor(KDR) | | | |  |
| SFRP1 | 6422 | secreted frizzled related protein 1(SFRP1) | | | |  |
| MAPK14 | 1432 | mitogen-activated protein kinase 14(MAPK14) | | | | |
| GSK3B | 2932 | glycogen synthase kinase 3 beta(GSK3B) | | | |  |
| ATP2A1 | 487 | ATPase sarcoplasmic/endoplasmic reticulum Ca2  + transporting 1(ATP2A1) | | | | |
| MAP2 | 4133 | microtubule associated protein 2(MAP2) | | | |  |
| PON1 | 5444 | paraoxonase 1(PON1) | | |  |  |
| ADRA1B | 147 | adrenoceptor alpha 1B(ADRA1B) | | | |  |
| FABP4 | 2167 | fatty acid binding protein 4(FABP4) | | | |  |
| ALOX5 | 240 | arachidonate 5-lipoxygenase(ALOX5) | | | |  |
| HTR2A | 3356 | 5-hydroxytryptamine receptor 2A(HTR2A) | | | |  |
| F2R | 2149 | coagulation factor II thrombin receptor(F2R) | | | | |
