# Supplementary material for: Systems Pharmacology and Verification of ShenFuHuang Formula in Zebrafish Model Reveal Multi-Scale Treatment Strategy for Septic Syndrome in COVID-19
Source: Front Pharmacol. 2020 Sep 15;11:584057. doi: 10.3389/fphar.2020.584057 (PMC7523021; doi:10.3389/fphar.2020.584057)
Supplement: Supplementary file 4 [file Table_4.docx]

**Supplementary Table S4**

**Node properties in Compound-Target network**

|  | | | | |  |  | |  | |  | |  | |  | |  | |  |
| --- | --- | --- | --- | --- | --- | --- | --- | --- | --- | --- | --- | --- | --- | --- | --- | --- | --- | --- |
| **Name** | **Node** | **Average Shortest PathLength** | **Betweenness Centrality** | **Closeness Centrality** | | | **Clustering Coefficient** | | **Degree** | | **Eccentricity** | | **Neighborhood Connectivity** | | **Stress** | | **Topological**  **Coefficient** | |
| MOL01 | Molecule | 2.892857 | 0.283858 | 0.345679 | | | 0 | | 24 | | 6 | | 5.791667 | | 58240 | | 0.228175 | |
| MOL02 | Molecule | 3.285714 | 0.079421 | 0.304348 | | | 0 | | 2 | | 5 | | 9.5 | | 16294 | | 0.5 | |
| MOL03 | Molecule | 3.392857 | 0.079047 | 0.294737 | | | 0 | | 17 | | 6 | | 6.823529 | | 23598 | | 0.323529 | |
| MOL04 | Molecule | 3.660714 | 0.079476 | 0.273171 | | | 0 | | 19 | | 7 | | 6.473684 | | 33300 | | 0.304094 | |
| MOL05 | Molecule | 3.392857 | 0.045435 | 0.294737 | | | 0 | | 16 | | 6 | | 7.8125 | | 17998 | | 0.358553 | |
| MOL06 | Molecule | 4.267857 | 0.003051 | 0.23431 | | | 0 | | 5 | | 7 | | 5.4 | | 580 | | 0.4 | |
| MOL07 | Molecule | 4 | 0.006271 | 0.25 | | | 0 | | 5 | | 8 | | 7.6 | | 3352 | | 0.55 | |
| MOL08 | Molecule | 3.767857 | 0.023341 | 0.265403 | | | 0 | | 13 | | 7 | | 7.923077 | | 13444 | | 0.384615 | |
| MOL09 | Molecule | 3.892857 | 0.006803 | 0.256881 | | | 0 | | 6 | | 7 | | 9.833333 | | 3862 | | 0.490741 | |
| MOL10 | Molecule | 3.785714 | 0.012049 | 0.264151 | | | 0 | | 12 | | 7 | | 8.583333 | | 2540 | | 0.421296 | |
| MOL11 | Molecule | 4.035714 | 5.26E-04 | 0.247788 | | | 0 | | 2 | | 7 | | 13 | | 154 | | 0.75 | |
| MOL12 | Molecule | 3.357143 | 0.04355 | 0.297872 | | | 0 | | 13 | | 6 | | 8.153846 | | 14918 | | 0.357692 | |
| MOL13 | Molecule | 3.946429 | 0.002841 | 0.253394 | | | 0 | | 5 | | 7 | | 10 | | 644 | | 0.5625 | |
| MOL14 | Molecule | 3.410714 | 0.03379 | 0.293194 | | | 0 | | 10 | | 6 | | 8.7 | | 11628 | | 0.385 | |
| MOL15 | Molecule | 4.089286 | 0 | 0.244541 | | | 0 | | 1 | | 7 | | 15 | | 0 | | 0 | |
| MOL16 | Molecule | 4.428571 | 0.011926 | 0.225806 | | | 0 | | 4 | | 8 | | 6.75 | | 4344 | | 0.522727 | |
| MOL17 | Molecule | 4.428571 | 0.024382 | 0.225806 | | | 0 | | 4 | | 8 | | 4.75 | | 4704 | | 0.340909 | |
| MOL18 | Molecule | 4.089286 | 0 | 0.244541 | | | 0 | | 1 | | 7 | | 15 | | 0 | | 0 | |
| MOL19 | Molecule | 4.035714 | 0.016234 | 0.247788 | | | 0 | | 6 | | 7 | | 9.833333 | | 8290 | | 0.401515 | |
| MOL20 | Molecule | 4.375 | 0.036398 | 0.228571 | | | 0 | | 3 | | 7 | | 5.333333 | | 4534 | | 0.393939 | |
| MOL21 | Molecule | 4.035714 | 0.008706 | 0.247788 | | | 0 | | 4 | | 7 | | 12 | | 2730 | | 0.458333 | |
| MOL22 | Molecule | 4.625 | 3.41E-04 | 0.216216 | | | 0 | | 2 | | 7 | | 12.5 | | 160 | | 0.766667 | |
| MOL23 | Molecule | 5.410714 | 4.14E-04 | 0.184818 | | | 0 | | 2 | | 9 | | 5.5 | | 56 | | 0.75 | |
| MOL24 | Molecule | 4.946429 | 0.00548 | 0.202166 | | | 0 | | 3 | | 9 | | 7.333333 | | 1184 | | 0.422222 | |
| MOL25 | Molecule | 4.625 | 0.003506 | 0.216216 | | | 0 | | 3 | | 7 | | 6.333333 | | 776 | | 0.533333 | |
| MOL26 | Molecule | 4.464286 | 0.00487 | 0.224 | | | 0 | | 3 | | 7 | | 8 | | 1236 | | 0.4375 | |
| MOL27 | Molecule | 4.446429 | 0.002907 | 0.2249 | | | 0 | | 3 | | 7 | | 11 | | 898 | | 0.588235 | |
| MOL28 | Molecule | 3.875 | 0.047852 | 0.258065 | | | 0 | | 5 | | 7 | | 7.4 | | 9260 | | 0.32 | |
| MOL29 | Molecule | 3.875 | 0.036427 | 0.258065 | | | 0 | | 6 | | 7 | | 10.5 | | 9116 | | 0.351852 | |
| MOL30 | Molecule | 4.464286 | 0.005771 | 0.224 | | | 0 | | 3 | | 7 | | 8.333333 | | 3952 | | 0.564103 | |
| MOL31 | Molecule | 4.464286 | 0.005771 | 0.224 | | | 0 | | 3 | | 7 | | 8.333333 | | 3952 | | 0.564103 | |
| MOL32 | Molecule | 4.464286 | 0.005771 | 0.224 | | | 0 | | 3 | | 7 | | 8.333333 | | 3952 | | 0.564103 | |
| MOL33 | Molecule | 4.303571 | 0.007711 | 0.232365 | | | 0 | | 6 | | 7 | | 9.5 | | 3992 | | 0.472222 | |
| MOL34 | Molecule | 3.982143 | 0.021974 | 0.251121 | | | 0 | | 7 | | 7 | | 10 | | 10180 | | 0.375 | |
| MOL35 | Molecule | 4.553571 | 0 | 0.219608 | | | 0 | | 1 | | 7 | | 13 | | 0 | | 0 | |
| MOL36 | Molecule | 4.303571 | 0.007711 | 0.232365 | | | 0 | | 6 | | 7 | | 9.5 | | 3992 | | 0.472222 | |
| MOL37 | Molecule | 4.339286 | 0.005144 | 0.230453 | | | 0 | | 5 | | 7 | | 10.2 | | 2558 | | 0.541176 | |
| MOL38 | Molecule | 4.339286 | 0.031571 | 0.230453 | | | 0 | | 6 | | 7 | | 6 | | 9710 | | 0.294118 | |
| MOL39 | Molecule | 4.553571 | 0 | 0.219608 | | | 0 | | 1 | | 7 | | 13 | | 0 | | 0 | |
| MOL40 | Molecule | 4.357143 | 0.005033 | 0.229508 | | | 0 | | 5 | | 7 | | 9.2 | | 3052 | | 0.5125 | |
| MOL41 | Molecule | 4.392857 | 0.007629 | 0.227642 | | | 0 | | 4 | | 7 | | 7.25 | | 4040 | | 0.390625 | |
| MOL42 | Molecule | 3.446429 | 0.152108 | 0.290155 | | | 0 | | 2 | | 6 | | 10 | | 54060 | | 0.5 | |
| MOL43 | Molecule | 5.053571 | 0.009616 | 0.19788 | | | 0 | | 3 | | 9 | | 6 | | 1048 | | 0.454545 | |
| MOL44 | Molecule | 4.482143 | 0.001221 | 0.223108 | | | 0 | | 2 | | 7 | | 11 | | 340 | | 0.625 | |
| MOL45 | Molecule | 3.696429 | 0.073595 | 0.270531 | | | 0 | | 9 | | 7 | | 7.444444 | | 15246 | | 0.257778 | |
| MOL46 | Molecule | 3.339286 | 0.167625 | 0.299465 | | | 0 | | 7 | | 5 | | 5.714286 | | 37552 | | 0.24812 | |
| MOL47 | Molecule | 4.071429 | 0.073531 | 0.245614 | | | 0 | | 7 | | 7 | | 2.142857 | | 11802 | | 0.190476 | |
| MOL48 | Molecule | 3.607143 | 0.121639 | 0.277228 | | | 0 | | 8 | | 7 | | 3.25 | | 13760 | | 0.140625 | |
| MOL49 | Molecule | 4 | 0.059683 | 0.25 | | | 0 | | 4 | | 7 | | 5.5 | | 10012 | | 0.409091 | |
| ACHE | Target | 3.258929 | 0.161296 | 0.306849 | | | 0 | | 7 | | 7 | | 12.42857 | | 57042 | | 0.394089 | |
| ADCY5 | Target | 4.294643 | 0.004398 | 0.232848 | | | 0 | | 2 | | 8 | | 7.5 | | 760 | | 0.590909 | |
| ADRA1B | Target | 3.848214 | 0.002064 | 0.259861 | | | 0 | | 3 | | 7 | | 14 | | 736 | | 0.565217 | |
| ADRB2 | Target | 3.616071 | 0.053293 | 0.276543 | | | 0 | | 5 | | 6 | | 11.8 | | 11016 | | 0.372414 | |
| AKR1B10 | Target | 4.633929 | 0.016925 | 0.2158 | | | 0 | | 7 | | 8 | | 4 | | 3446 | | 0.272727 | |
| ALOX5 | Target | 4.6875 | 0 | 0.213333 | | | 0 | | 1 | | 8 | | 9 | | 0 | | 0 | |
| ATP2A1 | Target | 4.294643 | 0.004398 | 0.232848 | | | 0 | | 2 | | 8 | | 7.5 | | 760 | | 0.590909 | |
| BCL2 | Target | 3.223214 | 0.143453 | 0.310249 | | | 0 | | 2 | | 6 | | 15.5 | | 32752 | | 0.5 | |
| BIRC2 | Target | 5.330357 | 0 | 0.187605 | | | 0 | | 1 | | 8 | | 6 | | 0 | | 0 | |
| CA2 | Target | 3.580357 | 0.013122 | 0.279302 | | | 0 | | 9 | | 7 | | 13.88889 | | 7814 | | 0.402778 | |
| CBR1 | Target | 3.866071 | 0.052041 | 0.258661 | | | 0 | | 2 | | 7 | | 6 | | 8102 | | 0.5 | |
| CCR6 | Target | 4.598214 | 0 | 0.217476 | | | 0 | | 1 | | 8 | | 8 | | 0 | | 0 | |
| CHRM2 | Target | 3.883929 | 0 | 0.257471 | | | 0 | | 1 | | 7 | | 24 | | 0 | | 0 | |
| CHRM3 | Target | 3.848214 | 0.002126 | 0.259861 | | | 0 | | 3 | | 7 | | 13 | | 734 | | 0.521739 | |
| CHRNA4 | Target | 5.0625 | 0 | 0.197531 | | | 0 | | 1 | | 8 | | 7 | | 0 | | 0 | |
| CHRNA7 | Target | 3.883929 | 0 | 0.257471 | | | 0 | | 1 | | 7 | | 24 | | 0 | | 0 | |
| CNR2 | Target | 5.008929 | 0.006003 | 0.199643 | | | 0 | | 5 | | 8 | | 3.6 | | 1438 | | 0.433333 | |
| CXCL8 | Target | 4.598214 | 0 | 0.217476 | | | 0 | | 1 | | 8 | | 8 | | 0 | | 0 | |
| CYP2A6 | Target | 5.0625 | 0 | 0.197531 | | | 0 | | 1 | | 8 | | 7 | | 0 | | 0 | |
| CYP3A4 | Target | 5.419643 | 0 | 0.184514 | | | 0 | | 1 | | 9 | | 4 | | 0 | | 0 | |
| EDNRA | Target | 4.169643 | 0.036442 | 0.239829 | | | 0 | | 10 | | 8 | | 5.8 | | 7926 | | 0.342857 | |
| EDNRB | Target | 4.258929 | 0.034658 | 0.234801 | | | 0 | | 11 | | 8 | | 4.909091 | | 8660 | | 0.279221 | |
| EGF | Target | 4.651786 | 0 | 0.214971 | | | 0 | | 1 | | 8 | | 19 | | 0 | | 0 | |
| ERG | Target | 3.705357 | 0.055689 | 0.26988 | | | 0 | | 9 | | 6 | | 6.111111 | | 16030 | | 0.340741 | |
| ESR1 | Target | 3.098214 | 0.210125 | 0.322767 | | | 0 | | 15 | | 6 | | 9.933333 | | 36214 | | 0.223333 | |
| F2 | Target | 3.5625 | 0.024782 | 0.280702 | | | 0 | | 11 | | 7 | | 11.36364 | | 10350 | | 0.334311 | |
| F2R | Target | 4.544643 | 0.003477 | 0.220039 | | | 0 | | 3 | | 8 | | 6.333333 | | 804 | | 0.410256 | |
| F7 | Target | 4.598214 | 3.17E-04 | 0.217476 | | | 0 | | 3 | | 8 | | 12.66667 | | 70 | | 0.614035 | |
| FABP4 | Target | 4.991071 | 0 | 0.200358 | | | 0 | | 1 | | 8 | | 4 | | 0 | | 0 | |
| FASN | Target | 4.383929 | 0 | 0.228106 | | | 0 | | 1 | | 7 | | 17 | | 0 | | 0 | |
| FKBP5 | Target | 3.6875 | 0.088658 | 0.271186 | | | 0 | | 14 | | 6 | | 5.214286 | | 23792 | | 0.263393 | |
| GLB1 | Target | 3.5625 | 0.199774 | 0.280702 | | | 0 | | 13 | | 6 | | 4.230769 | | 60796 | | 0.215385 | |
| GSK3B | Target | 3.598214 | 0.008961 | 0.277916 | | | 0 | | 8 | | 7 | | 15.5 | | 6258 | | 0.453125 | |
| HPSE | Target | 3.741071 | 0.070804 | 0.267303 | | | 0 | | 8 | | 6 | | 4 | | 11966 | | 0.230769 | |
| HSPB1 | Target | 4.026786 | 0.002408 | 0.248337 | | | 0 | | 2 | | 6 | | 8 | | 598 | | 0.636364 | |
| HTR2A | Target | 3.883929 | 0 | 0.257471 | | | 0 | | 1 | | 7 | | 24 | | 0 | | 0 | |
| IFNG | Target | 5.383929 | 1.32E-04 | 0.185738 | | | 0 | | 2 | | 9 | | 4 | | 8 | | 0.75 | |
| IGHG1 | Target | 4.223214 | 6.29E-04 | 0.236786 | | | 0 | | 3 | | 7 | | 17.33333 | | 720 | | 0.742424 | |
| JUN | Target | 3.866071 | 3.86E-04 | 0.258661 | | | 0 | | 2 | | 7 | | 18 | | 202 | | 0.73913 | |
| KDR | Target | 4.616071 | 7.84E-05 | 0.216634 | | | 0 | | 2 | | 8 | | 16 | | 26 | | 0.789474 | |
| MAP2 | Target | 3.883929 | 0 | 0.257471 | | | 0 | | 1 | | 7 | | 24 | | 0 | | 0 | |
| MAPK14 | Target | 3.633929 | 0.006007 | 0.275184 | | | 0 | | 6 | | 7 | | 16.83333 | | 4172 | | 0.494792 | |
| MMP1 | Target | 4.651786 | 0 | 0.214971 | | | 0 | | 1 | | 8 | | 19 | | 0 | | 0 | |
| NOS1 | Target | 4.258929 | 0.034776 | 0.234801 | | | 0 | | 2 | | 6 | | 5 | | 5218 | | 0.5 | |
| NOS2 | Target | 3.5625 | 0.021574 | 0.280702 | | | 0 | | 9 | | 7 | | 14.22222 | | 9978 | | 0.400673 | |
| NOS3 | Target | 4.294643 | 5.80E-04 | 0.232848 | | | 0 | | 3 | | 7 | | 12.66667 | | 222 | | 0.648148 | |
| NR3C2 | Target | 3.401786 | 0.085163 | 0.293963 | | | 0 | | 4 | | 6 | | 5.75 | | 17714 | | 0.339286 | |
| PIK3CG | Target | 3.633929 | 0.006407 | 0.275184 | | | 0 | | 6 | | 7 | | 15.5 | | 3572 | | 0.453125 | |
| PIM1 | Target | 3.580357 | 0.011838 | 0.279302 | | | 0 | | 9 | | 7 | | 13.55556 | | 6804 | | 0.392361 | |
| PLAA | Target | 4.294643 | 0.004398 | 0.232848 | | | 0 | | 2 | | 8 | | 7.5 | | 760 | | 0.590909 | |
| PON1 | Target | 3.883929 | 0 | 0.257471 | | | 0 | | 1 | | 7 | | 24 | | 0 | | 0 | |
| PPARG | Target | 3.598214 | 0.010623 | 0.277916 | | | 0 | | 8 | | 7 | | 14.875 | | 6656 | | 0.433594 | |
| PPP3CA | Target | 4.776786 | 0.010999 | 0.209346 | | | 0 | | 5 | | 8 | | 3.2 | | 2714 | | 0.44 | |
| PRSS1 | Target | 3.633929 | 0.006444 | 0.275184 | | | 0 | | 6 | | 7 | | 17 | | 4686 | | 0.5 | |
| PTGS1 | Target | 3.598214 | 0.00948 | 0.277916 | | | 0 | | 8 | | 7 | | 14.5 | | 5380 | | 0.421875 | |
| PTGS2 | Target | 3.491071 | 0.063389 | 0.286445 | | | 0 | | 12 | | 7 | | 11.91667 | | 18176 | | 0.321078 | |
| REN | Target | 4.776786 | 0.008586 | 0.209346 | | | 0 | | 7 | | 8 | | 5.857143 | | 2082 | | 0.485714 | |
| SCARB1 | Target | 4.330357 | 0 | 0.230928 | | | 0 | | 1 | | 6 | | 7 | | 0 | | 0 | |
| SCN5A | Target | 3.741071 | 0.004952 | 0.267303 | | | 0 | | 5 | | 7 | | 14.2 | | 2516 | | 0.488889 | |
| SFRP1 | Target | 4.830357 | 0.003773 | 0.207024 | | | 0 | | 6 | | 8 | | 5.666667 | | 950 | | 0.583333 | |
| SMAD3 | Target | 4.598214 | 0 | 0.217476 | | | 0 | | 1 | | 8 | | 8 | | 0 | | 0 | |
| SPHK1 | Target | 4.455357 | 0.033572 | 0.224449 | | | 0 | | 6 | | 8 | | 3.166667 | | 5324 | | 0.270833 | |
| TNF | Target | 4.1875 | 0.007564 | 0.238806 | | | 0 | | 4 | | 7 | | 11 | | 1954 | | 0.434783 | |
| TP53 | Target | 4.258929 | 3.02E-04 | 0.234801 | | | 0 | | 2 | | 7 | | 18 | | 348 | | 0.809524 | |

**Node properties in Target-Disease network**

| **Name** | **Node type** | **Average Shortest Path Length** | **Betweenness Centrality** | **Closeness Centrality** | **Clustering Coefficient** | **Degree** | **Eccentricity** | **Neighborhood Connectivity** | **Radiality** | **Stress** | **Topological Coefficient** |
| --- | --- | --- | --- | --- | --- | --- | --- | --- | --- | --- | --- |
| Inflammation | Disease | 1.63636364 | 0.553768 | 0.611111 | 0.042236 | 72 | 3 | 5.928571 | 0.893939 | 30730 | 0.041201 |
| Immune system disease | Disease | 1.88484848 | 0.323688 | 0.530547 | 0.018357 | 46 | 4 | 6 | 0.852525 | 34902 | 0.047259 |
| Bacterial infection or mycosis | Disease | 2.36969697 | 0.053978 | 0.421995 | 0.075 | 16 | 4 | 9.6875 | 0.771717 | 7998 | 0.102335 |
| Metabolic disease | Disease | 2.34545455 | 0.094009 | 0.426357 | 0.058333 | 16 | 4 | 9.1875 | 0.775758 | 11192 | 0.094429 |
| Arthritis, Rheumatoid | Disease | 2.27272727 | 0.049655 | 0.44 | 0.076923 | 13 | 4 | 17.30769 | 0.787879 | 6668 | 0.163555 |
| Heart Failure | Disease | 2.24848485 | 0.049467 | 0.444744 | 0.089744 | 13 | 4 | 16.38462 | 0.791919 | 6388 | 0.149533 |
| Diabetes Mellitus, Type 2 | Disease | 2.23030303 | 0.053178 | 0.44837 | 0.090909 | 12 | 3 | 17.66667 | 0.794949 | 6802 | 0.156818 |
| Colitis | Disease | 2.30909091 | 0.00994 | 0.433071 | 0.194444 | 9 | 4 | 24.77778 | 0.781818 | 3144 | 0.232704 |
| Dermatitis, Contact | Disease | 2.25454545 | 0.042224 | 0.443548 | 0.083333 | 9 | 3 | 17.66667 | 0.790909 | 4358 | 0.156983 |
| Brain Injuries | Disease | 2.2969697 | 0.033856 | 0.435356 | 0.142857 | 8 | 4 | 22.375 | 0.783838 | 3618 | 0.207547 |
| Drug-Induced Liver Injury | Disease | 2.62424242 | 0.021966 | 0.381062 | 0 | 8 | 5 | 15.625 | 0.729293 | 3776 | 0.218284 |
| Viral disease | Disease | 2.47878788 | 0.010728 | 0.403423 | 0.142857 | 8 | 4 | 15.125 | 0.753535 | 1932 | 0.173529 |
| Dermatitis, Allergic Contact | Disease | 2.33333333 | 0.013275 | 0.428571 | 0.190476 | 7 | 4 | 27.85714 | 0.777778 | 2610 | 0.267684 |
| Hepatitis C | Disease | 2.32727273 | 0.024935 | 0.429688 | 0.142857 | 7 | 4 | 23.28571 | 0.778788 | 2804 | 0.221914 |
| Acute Kidney Injury | Disease | 2.36363636 | 0.013575 | 0.423077 | 0.2 | 6 | 4 | 27.16667 | 0.772727 | 1724 | 0.268333 |
| Arthritis, Juvenile | Disease | 2.37575758 | 0.032277 | 0.420918 | 0.066667 | 6 | 4 | 20.66667 | 0.770707 | 2122 | 0.208333 |
| Inflammatory Bowel Diseases | Disease | 2.32121212 | 0.015453 | 0.430809 | 0.266667 | 6 | 4 | 29.16667 | 0.779798 | 2092 | 0.273585 |
| Kidney Failure, Chronic | Disease | 2.36969697 | 0.021228 | 0.421995 | 0.133333 | 6 | 4 | 21.66667 | 0.771717 | 1836 | 0.215986 |
| Pancreatitis | Disease | 2.38181818 | 0.028452 | 0.419847 | 0.133333 | 6 | 4 | 21.83333 | 0.769697 | 1896 | 0.222222 |
| Arthritis, Psoriatic | Disease | 2.35757576 | 0.002456 | 0.424165 | 0.4 | 5 | 4 | 34.2 | 0.773737 | 912 | 0.328846 |
| Colitis, Ulcerative | Disease | 2.52121212 | 0.014747 | 0.396635 | 0.2 | 5 | 4 | 23.2 | 0.746465 | 974 | 0.281481 |
| Diabetes Mellitus, Experimental | Disease | 2.78181818 | 0.006535 | 0.359477 | 0 | 5 | 5 | 12.8 | 0.70303 | 1090 | 0.240816 |
| Pulmonary Disease | Disease | 2.53939394 | 0.012538 | 0.393795 | 0.3 | 5 | 4 | 22 | 0.743434 | 652 | 0.275949 |
| Arthritis, Experimental | Disease | 2.68484848 | 0.002965 | 0.372461 | 0 | 4 | 5 | 26.25 | 0.719192 | 848 | 0.388462 |
| Arthritis, Infectious | Disease | 2.49090909 | 4.72E-04 | 0.40146 | 0.5 | 4 | 4 | 35.5 | 0.751515 | 266 | 0.403409 |
| Chlamydia Infections | Disease | 2.93333333 | 7.98E-04 | 0.340909 | 0 | 4 | 5 | 20 | 0.677778 | 368 | 0.365385 |
| Dermatitis, Atopic | Disease | 2.39393939 | 0.00546 | 0.417722 | 0.333333 | 4 | 4 | 35.5 | 0.767677 | 698 | 0.359694 |
| Diabetes Mellitus | Disease | 2.50909091 | 0.002909 | 0.398551 | 0.333333 | 4 | 4 | 25 | 0.748485 | 486 | 0.305556 |
| Hepatitis B | Disease | 2.39393939 | 0.003934 | 0.417722 | 0.333333 | 4 | 4 | 36 | 0.767677 | 702 | 0.364796 |
| HIV Infections | Disease | 2.53939394 | 0.014134 | 0.393795 | 0.166667 | 4 | 4 | 24.5 | 0.743434 | 802 | 0.303797 |
| Metabolic Diseases | Disease | 2.40606061 | 0.012509 | 0.415617 | 0.333333 | 4 | 4 | 31.25 | 0.765657 | 662 | 0.322917 |
| Osteoarthritis | Disease | 2.71515152 | 0.006277 | 0.368304 | 0 | 4 | 5 | 18 | 0.714141 | 1090 | 0.309091 |
| Peritonitis | Disease | 2.39393939 | 0.004656 | 0.417722 | 0.333333 | 4 | 4 | 35.75 | 0.767677 | 752 | 0.362245 |
| Pneumonia | Disease | 2.50909091 | 0.002838 | 0.398551 | 0.333333 | 4 | 4 | 32.25 | 0.748485 | 492 | 0.376471 |
| Renal Insufficiency | Disease | 2.50909091 | 6.10E-04 | 0.398551 | 0.5 | 4 | 4 | 29.5 | 0.748485 | 188 | 0.347059 |
| Disease Progression | Disease | 2.85454545 | 0.006824 | 0.350318 | 0 | 3 | 5 | 17.33333 | 0.690909 | 1226 | 0.362963 |
| Glomerulonephritis | Disease | 2.84242424 | 0.00697 | 0.351812 | 0 | 3 | 5 | 18 | 0.692929 | 1258 | 0.361702 |
| Hepatitis | Disease | 2.78787879 | 0.00137 | 0.358696 | 0 | 3 | 5 | 24 | 0.70202 | 238 | 0.433962 |
| Leukemia | Disease | 2.84848485 | 0.016564 | 0.351064 | 0 | 3 | 5 | 16.33333 | 0.691919 | 1222 | 0.333333 |
| Neurotoxicity Syndromes | Disease | 2.84242424 | 0.012907 | 0.351812 | 0 | 3 | 5 | 17.66667 | 0.692929 | 1270 | 0.35461 |
| Shock, Septic | Disease | 3.03636364 | 5.05E-04 | 0.329341 | 0 | 3 | 5 | 20.33333 | 0.660606 | 216 | 0.449612 |
| Acute Lung Injury | Disease | 2.61818182 | 0 | 0.381944 | 1 | 2 | 4 | 39 | 0.730303 | 0 | 0.549296 |
| Anemia, Aplastic | Disease | 2.8 | 2.11E-04 | 0.357143 | 0 | 2 | 5 | 35 | 0.7 | 150 | 0.641509 |
| Appendicitis | Disease | 2.8 | 2.11E-04 | 0.357143 | 0 | 2 | 5 | 35 | 0.7 | 150 | 0.641509 |
| Arteritis | Disease | 2.58181818 | 0.001326 | 0.387324 | 0 | 2 | 4 | 38 | 0.736364 | 226 | 0.513889 |
| Brain Edema | Disease | 2.56363636 | 0 | 0.390071 | 1 | 2 | 4 | 51 | 0.739394 | 0 | 0.64557 |
| Brain Ischemia | Disease | 2.86666667 | 1.82E-04 | 0.348837 | 0 | 2 | 5 | 28 | 0.688889 | 62 | 0.6 |
| Bronchiectasis | Disease | 2.56363636 | 0 | 0.390071 | 1 | 2 | 4 | 51 | 0.739394 | 0 | 0.64557 |
| Cardiomyopathy, Dilated, 1E | Disease | 2.61818182 | 0.012121 | 0.381944 | 0 | 2 | 4 | 35.5 | 0.730303 | 448 | 0.5 |
| Cardiovirus Infections | Disease | 2.56363636 | 0 | 0.390071 | 1 | 2 | 4 | 51 | 0.739394 | 0 | 0.64557 |
| Cholangitis, Sclerosing | Disease | 2.6 | 0 | 0.384615 | 1 | 2 | 4 | 41.5 | 0.733333 | 0 | 0.560811 |
| Coxsackievirus Infections | Disease | 2.56363636 | 0 | 0.390071 | 1 | 2 | 4 | 51 | 0.739394 | 0 | 0.64557 |
| Dermatitis, Irritant | Disease | 2.61818182 | 0.003827 | 0.381944 | 0 | 2 | 4 | 36 | 0.730303 | 308 | 0.507246 |
| Dermatomyositis | Disease | 2.75151515 | 2.58E-04 | 0.363436 | 0 | 2 | 5 | 39 | 0.708081 | 212 | 0.633333 |
| Diabetes Mellitus, Type 1 | Disease | 2.86666667 | 0.012121 | 0.348837 | 0 | 2 | 5 | 23.5 | 0.688889 | 992 | 0.5 |
| Diabetic Nephropathies | Disease | 3.35151515 | 0.012121 | 0.298373 | 0 | 2 | 5 | 8.5 | 0.608081 | 1244 | 0.5 |
| Disease Models | Disease | 2.86666667 | 0.012121 | 0.348837 | 0 | 2 | 5 | 23.5 | 0.688889 | 992 | 0.5 |
| Disease Models, Animal | Disease | 2.61818182 | 0.002154 | 0.381944 | 0 | 2 | 4 | 36.5 | 0.730303 | 276 | 0.514493 |
| Endotoxemia | Disease | 2.86060606 | 0.001051 | 0.349576 | 0 | 2 | 5 | 25 | 0.689899 | 206 | 0.521739 |
| Enterocolitis, Necrotizing | Disease | 3.27272727 | 1.09E-04 | 0.305556 | 0 | 2 | 5 | 14.5 | 0.621212 | 32 | 0.642857 |
| Esophagitis, Peptic | Disease | 2.61212121 | 0 | 0.382831 | 1 | 2 | 4 | 40 | 0.731313 | 0 | 0.555556 |
| Eye Infections, Bacterial | Disease | 2.61212121 | 0 | 0.382831 | 1 | 2 | 4 | 40 | 0.731313 | 0 | 0.555556 |
| Genetic Predisposition to Disease | Disease | 2.61818182 | 0.003987 | 0.381944 | 0 | 2 | 4 | 36 | 0.730303 | 320 | 0.507246 |
| Glomerulonephritis, IGA | Disease | 2.61818182 | 0.012121 | 0.381944 | 0 | 2 | 4 | 35.5 | 0.730303 | 448 | 0.5 |
| Hepatitis, Alcoholic | Disease | 2.75151515 | 2.58E-04 | 0.363436 | 0 | 2 | 5 | 39 | 0.708081 | 212 | 0.633333 |
| Hepatitis, Autoimmune | Disease | 2.8 | 2.11E-04 | 0.357143 | 0 | 2 | 5 | 35 | 0.7 | 150 | 0.641509 |
| Hyperalgesia | Disease | 2.75151515 | 2.58E-04 | 0.363436 | 0 | 2 | 5 | 39 | 0.708081 | 212 | 0.633333 |
| Immune Complex Diseases | Disease | 2.56363636 | 0 | 0.390071 | 1 | 2 | 4 | 47 | 0.739394 | 0 | 0.594937 |
| Immune System Diseases | Disease | 2.61818182 | 0.003862 | 0.381944 | 0 | 2 | 4 | 36 | 0.730303 | 320 | 0.507246 |
| Infection | Disease | 2.75151515 | 2.58E-04 | 0.363436 | 0 | 2 | 5 | 39 | 0.708081 | 212 | 0.633333 |
| Keratitis | Disease | 3.31515152 | 6.49E-05 | 0.301645 | 0 | 2 | 5 | 13 | 0.614141 | 12 | 0.6 |
| Leukemia, Lymphocytic, Chronic, B-Cell | Disease | 2.86666667 | 1.82E-04 | 0.348837 | 0 | 2 | 5 | 28 | 0.688889 | 62 | 0.6 |
| Leukemia, Myeloid, Acute | Disease | 2.60606061 | 0.00753 | 0.383721 | 0 | 2 | 4 | 36 | 0.732323 | 332 | 0.5 |
| Liver Diseases | Disease | 3.27272727 | 1.09E-04 | 0.305556 | 0 | 2 | 5 | 14.5 | 0.621212 | 32 | 0.642857 |
| Lung Diseases | Disease | 2.86666667 | 0.012121 | 0.348837 | 0 | 2 | 5 | 23.5 | 0.688889 | 992 | 0.5 |
| Lung Injury | Disease | 2.56363636 | 0 | 0.390071 | 1 | 2 | 4 | 51 | 0.739394 | 0 | 0.64557 |
| Lymphoma, Large B-Cell, Diffuse | Disease | 2.61818182 | 0.012121 | 0.381944 | 0 | 2 | 4 | 35.5 | 0.730303 | 448 | 0.5 |
| Lymphoma, T-Cell | Disease | 3.29090909 | 9.85E-04 | 0.303867 | 0 | 2 | 5 | 10 | 0.618182 | 106 | 0.5 |
| Lymphoma, T-Cell, Cutaneous | Disease | 2.85454545 | 0.001641 | 0.350318 | 0 | 2 | 5 | 25 | 0.690909 | 272 | 0.521739 |
| Mitochondrial Diseases | Disease | 2.6 | 0 | 0.384615 | 1 | 2 | 4 | 41.5 | 0.733333 | 0 | 0.560811 |
| Multiple Organ Failure | Disease | 2.61212121 | 0.001526 | 0.382831 | 0 | 2 | 4 | 37 | 0.731313 | 244 | 0.514286 |
| Muscular Dystrophies | Disease | 3.32727273 | 0.012121 | 0.300546 | 0 | 2 | 5 | 8.5 | 0.612121 | 1168 | 0.5 |
| Mycoplasma Infections | Disease | 3.27272727 | 1.09E-04 | 0.305556 | 0 | 2 | 5 | 14.5 | 0.621212 | 32 | 0.642857 |
| Myocardial Reperfusion Injury | Disease | 3.31515152 | 3.43E-04 | 0.301645 | 0 | 2 | 5 | 10 | 0.614141 | 74 | 0.5625 |
| Myocarditis | Disease | 2.56363636 | 0 | 0.390071 | 1 | 2 | 4 | 51 | 0.739394 | 0 | 0.64557 |
| Myositis | Disease | 3.32727273 | 0.012121 | 0.300546 | 0 | 2 | 5 | 8.5 | 0.612121 | 1168 | 0.5 |
| Necrosis | Disease | 2.61818182 | 0.001127 | 0.381944 | 0 | 2 | 4 | 37 | 0.730303 | 220 | 0.521739 |
| Osteopetrosis with renal tubular acidosis | Disease | 2.61818182 | 0.012121 | 0.381944 | 0 | 2 | 4 | 35.5 | 0.730303 | 448 | 0.5 |
| Pain | Disease | 2.56363636 | 0 | 0.390071 | 1 | 2 | 4 | 51 | 0.739394 | 0 | 0.64557 |
| Pancreatitis, Chronic | Disease | 2.61818182 | 0.003862 | 0.381944 | 0 | 2 | 4 | 36 | 0.730303 | 320 | 0.507246 |
| Peripheral Nervous System Diseases | Disease | 2.86666667 | 0.001331 | 0.348837 | 0 | 2 | 5 | 24.5 | 0.688889 | 212 | 0.522222 |
| Pleurisy | Disease | 2.56363636 | 0 | 0.390071 | 1 | 2 | 4 | 51 | 0.739394 | 0 | 0.64557 |
| Pneumonia, Pneumococcal | Disease | 2.61212121 | 0 | 0.382831 | 1 | 2 | 4 | 40 | 0.731313 | 0 | 0.555556 |
| Polymyositis | Disease | 2.56363636 | 0 | 0.390071 | 1 | 2 | 4 | 51 | 0.739394 | 0 | 0.64557 |
| Pyelonephritis | Disease | 2.61818182 | 0.002154 | 0.381944 | 0 | 2 | 4 | 36.5 | 0.730303 | 276 | 0.514493 |
| Respiratory Syncytial Virus Infections | Disease | 2.56363636 | 0 | 0.390071 | 1 | 2 | 4 | 47 | 0.739394 | 0 | 0.594937 |
| Respiratory Tract Diseases | Disease | 2.8 | 2.11E-04 | 0.357143 | 0 | 2 | 5 | 35 | 0.7 | 150 | 0.641509 |
| Salmonella Infections, Animal | Disease | 3.22424242 | 7.10E-05 | 0.31015 | 0 | 2 | 5 | 16 | 0.629293 | 20 | 0.555556 |
| Staphylococcal Infections | Disease | 3.31515152 | 6.49E-05 | 0.301645 | 0 | 2 | 5 | 13 | 0.614141 | 12 | 0.6 |
| Vascular System Injuries | Disease | 3.0969697 | 1.53E-04 | 0.322896 | 0 | 2 | 5 | 24 | 0.650505 | 80 | 0.605263 |
| Ventricular Dysfunction, Left | Disease | 2.86666667 | 0.001906 | 0.348837 | 0 | 2 | 5 | 24 | 0.688889 | 214 | 0.511111 |
| TNF | Target | 2.22424242 | 0.067298 | 0.449591 | 0.044355 | 32 | 4 | 6.75 | 0.79596 | 10814 | 0.067383 |
| IFNG | Target | 2.32121212 | 0.041562 | 0.430809 | 0.050725 | 24 | 4 | 7.25 | 0.779798 | 6754 | 0.078125 |
| NOS2 | Target | 2.46060606 | 0.021708 | 0.406404 | 0.102564 | 13 | 4 | 10.30769 | 0.756566 | 4014 | 0.121951 |
| CXCL8 | Target | 2.51515152 | 0.011087 | 0.39759 | 0.155556 | 10 | 4 | 10.8 | 0.747475 | 1358 | 0.135897 |
| PTGS2 | Target | 2.43030303 | 0.012462 | 0.411471 | 0.133333 | 10 | 4 | 13.7 | 0.761616 | 1972 | 0.148889 |
| PPARG | Target | 2.52121212 | 0.003665 | 0.396635 | 0.214286 | 8 | 4 | 13.375 | 0.746465 | 416 | 0.165625 |
| Sepsis | Target | 2.30909091 | 0.010304 | 0.433071 | 0.238095 | 7 | 4 | 29.28571 | 0.781818 | 2968 | 0.272363 |
| PON1 | Target | 3.07878788 | 0.003647 | 0.324803 | 0 | 6 | 4 | 5.5 | 0.653535 | 572 | 0.236842 |
| ALOX5 | Target | 3.2 | 7.62E-04 | 0.3125 | 0 | 4 | 5 | 6.5 | 0.633333 | 128 | 0.392857 |
| BCL2 | Target | 3.12121212 | 8.92E-04 | 0.320388 | 0 | 4 | 4 | 7.5 | 0.646465 | 114 | 0.382353 |
| CYP2A6 | Target | 3.22424242 | 8.32E-04 | 0.31015 | 0 | 4 | 5 | 5.5 | 0.629293 | 174 | 0.375 |
| NOS3 | Target | 3.15757576 | 0.00171 | 0.316699 | 0 | 4 | 4 | 6.5 | 0.640404 | 462 | 0.392857 |
| TP53 | Target | 3.23030303 | 0.001356 | 0.309568 | 0 | 4 | 5 | 3.75 | 0.628283 | 318 | 0.305556 |
| CCR6 | Target | 3.23030303 | 3.13E-04 | 0.309568 | 0 | 3 | 5 | 7.333333 | 0.628283 | 40 | 0.487179 |
| GSK3B | Target | 3.32121212 | 1.14E-04 | 0.301095 | 0 | 3 | 5 | 4.666667 | 0.613131 | 14 | 0.611111 |
| HSPB1 | Target | 3.12121212 | 9.18E-04 | 0.320388 | 0 | 3 | 4 | 8.666667 | 0.646465 | 90 | 0.403509 |
| JUN | Target | 3.23030303 | 5.80E-04 | 0.309568 | 0 | 3 | 5 | 5.333333 | 0.628283 | 68 | 0.433333 |
| PIM1 | Target | 3.3030303 | 1.40E-04 | 0.302752 | 0 | 3 | 5 | 3.666667 | 0.616162 | 10 | 0.444444 |
| CBR1 | Target | 3.35151515 | 9.86E-05 | 0.298373 | 0 | 2 | 5 | 4 | 0.608081 | 6 | 0.6 |
| CNR2 | Target | 3.36363636 | 9.86E-05 | 0.297297 | 0 | 2 | 5 | 4 | 0.606061 | 6 | 0.6 |
| EGF | Target | 3.35757576 | 1.36E-04 | 0.297834 | 0 | 2 | 5 | 4 | 0.607071 | 8 | 0.6 |
| ERG | Target | 3.38181818 | 1.48E-04 | 0.295699 | 0 | 2 | 5 | 2.5 | 0.60303 | 4 | 0.5 |
| ESR1 | Target | 3.81212121 | 6.16E-05 | 0.262321 | 0 | 2 | 6 | 3 | 0.531313 | 6 | 0.666667 |
| F2R | Target | 2.61818182 | 0.001153 | 0.381944 | 0 | 2 | 4 | 36.5 | 0.730303 | 144 | 0.514493 |
| FASN | Target | 3.28484848 | 2.22E-04 | 0.304428 | 0 | 2 | 5 | 7 | 0.619192 | 26 | 0.545455 |
| GLB1 | Target | 3.22424242 | 9.27E-05 | 0.31015 | 0 | 2 | 4 | 6.5 | 0.629293 | 10 | 0.611111 |
| NR3C2 | Target | 3.23030303 | 2.87E-04 | 0.309568 | 0 | 2 | 5 | 7.5 | 0.628283 | 58 | 0.541667 |
| PRSS1 | Target | 3.36363636 | 9.86E-05 | 0.297297 | 0 | 2 | 5 | 4 | 0.606061 | 6 | 0.6 |
| PTGS1 | Target | 3.12727273 | 2.64E-04 | 0.319767 | 0 | 2 | 4 | 13 | 0.645455 | 60 | 0.6 |
| REN | Target | 3.20606061 | 2.22E-04 | 0.311909 | 0 | 2 | 5 | 9 | 0.632323 | 66 | 0.571429 |
| SCARB1 | Target | 3.27878788 | 1.27E-04 | 0.304991 | 0 | 2 | 5 | 6.5 | 0.620202 | 16 | 0.611111 |
| SMAD3 | Target | 3.29090909 | 1.26E-04 | 0.303867 | 0 | 2 | 5 | 5 | 0.618182 | 8 | 0.571429 |
| ACHE | Target | 4.32121212 | 0 | 0.231417 | 0 | 1 | 6 | 2 | 0.446465 | 0 | 0 |
| ADCY5 | Target | 3.22424242 | 0 | 0.31015 | 0 | 1 | 4 | 12 | 0.629293 | 0 | 0 |
| ADRA1B | Target | 3.83636364 | 0 | 0.260664 | 0 | 1 | 6 | 3 | 0.527273 | 0 | 0 |
| ADRB2 | Target | 3.86060606 | 0 | 0.259027 | 0 | 1 | 6 | 2 | 0.523232 | 0 | 0 |
| AKR1B10 | Target | 3.24848485 | 0 | 0.307836 | 0 | 1 | 4 | 9 | 0.625253 | 0 | 0 |
| ATP2A1 | Target | 3.24242424 | 0 | 0.308411 | 0 | 1 | 5 | 13 | 0.626263 | 0 | 0 |
| BIRC2 | Target | 3.53333333 | 0 | 0.283019 | 0 | 1 | 5 | 4 | 0.577778 | 0 | 0 |
| CA2 | Target | 3.61212121 | 0 | 0.276846 | 0 | 1 | 5 | 2 | 0.564646 | 0 | 0 |
| CHRM2 | Target | 3.29090909 | 0 | 0.303867 | 0 | 1 | 5 | 8 | 0.618182 | 0 | 0 |
| CHRM3 | Target | 3.61818182 | 0 | 0.276382 | 0 | 1 | 6 | 8 | 0.563636 | 0 | 0 |
| CHRNA4 | Target | 2.63030303 | 0 | 0.380184 | 0 | 1 | 4 | 70 | 0.728283 | 0 | 0 |
| CHRNA7 | Target | 3.29090909 | 0 | 0.303867 | 0 | 1 | 5 | 8 | 0.618182 | 0 | 0 |
| CYP3A4 | Target | 3.32121212 | 0 | 0.301095 | 0 | 1 | 5 | 7 | 0.613131 | 0 | 0 |
| EDNRA | Target | 3.22424242 | 0 | 0.31015 | 0 | 1 | 4 | 12 | 0.629293 | 0 | 0 |
| EDNRB | Target | 3.22424242 | 0 | 0.31015 | 0 | 1 | 4 | 12 | 0.629293 | 0 | 0 |
| F2 | Target | 3.26666667 | 0 | 0.306122 | 0 | 1 | 5 | 13 | 0.622222 | 0 | 0 |
| F7 | Target | 3.86060606 | 0 | 0.259027 | 0 | 1 | 6 | 2 | 0.523232 | 0 | 0 |
| FABP4 | Target | 3.24848485 | 0 | 0.307836 | 0 | 1 | 4 | 9 | 0.625253 | 0 | 0 |
| FKBP5 | Target | 3.26666667 | 0 | 0.306122 | 0 | 1 | 5 | 13 | 0.622222 | 0 | 0 |
| HPSE | Target | 3.61212121 | 0 | 0.276846 | 0 | 1 | 5 | 2 | 0.564646 | 0 | 0 |
| HTR2A | Target | 3.4 | 0 | 0.294118 | 0 | 1 | 5 | 4 | 0.6 | 0 | 0 |
| IGHG1 | Target | 4.32121212 | 0 | 0.231417 | 0 | 1 | 6 | 2 | 0.446465 | 0 | 0 |
| KDR | Target | 4.34545455 | 0 | 0.230126 | 0 | 1 | 6 | 2 | 0.442424 | 0 | 0 |
| MAP2 | Target | 3.36969697 | 0 | 0.296763 | 0 | 1 | 5 | 6 | 0.605051 | 0 | 0 |
| MAPK14 | Target | 3.84242424 | 0 | 0.260252 | 0 | 1 | 6 | 3 | 0.526263 | 0 | 0 |
| MMP1 | Target | 3.53333333 | 0 | 0.283019 | 0 | 1 | 5 | 5 | 0.577778 | 0 | 0 |
| NOS1 | Target | 3.86060606 | 0 | 0.259027 | 0 | 1 | 6 | 2 | 0.523232 | 0 | 0 |
| PIK3CG | Target | 3.61212121 | 0 | 0.276846 | 0 | 1 | 5 | 2 | 0.564646 | 0 | 0 |
| PLAA | Target | 2.63030303 | 0 | 0.380184 | 0 | 1 | 4 | 70 | 0.728283 | 0 | 0 |
| PPP3CA | Target | 3.37575758 | 0 | 0.29623 | 0 | 1 | 5 | 6 | 0.60404 | 0 | 0 |
| SCN5A | Target | 3.61212121 | 0 | 0.276846 | 0 | 1 | 5 | 2 | 0.564646 | 0 | 0 |
| SFRP1 | Target | 3.31515152 | 0 | 0.301645 | 0 | 1 | 5 | 6 | 0.614141 | 0 | 0 |
| SPHK1 | Target | 3.51515152 | 0 | 0.284483 | 0 | 1 | 5 | 5 | 0.580808 | 0 | 0 |

**Node properties in Target-Pathway network**

| **Name** | **Node** | **Average Shortest Path Length** | **Betweenness Centrality** | **Closeness Centrality** | **Clustering Coefficient** | **Degree** | **Eccentricity** | **Neighborhood Connectivity** | **Radiality** | **Stress** | **Topological**  **Coefficient** |
| --- | --- | --- | --- | --- | --- | --- | --- | --- | --- | --- | --- |
| Calcium signaling pathway | Pathway | 2.677778 | 0.084137 | 0.373444 | 0 | 15 | 5 | 3.933333 | 0.72037 | 8620 | 0.117333 |
| Pathways in cancer | Pathway | 2.122222 | 0.121301 | 0.471204 | 0 | 17 | 3 | 10.23529 | 0.812963 | 14740 | 0.205229 |
| VEGF signaling pathway | Pathway | 2.388889 | 0.034772 | 0.418605 | 0 | 8 | 4 | 10.875 | 0.768519 | 3952 | 0.235119 |
| cGMP-PKG signaling pathway | Pathway | 2.566667 | 0.043788 | 0.38961 | 0 | 9 | 5 | 7.777778 | 0.738889 | 5834 | 0.188272 |
| T cell receptor signaling pathway | Pathway | 2.433333 | 0.008865 | 0.410959 | 0 | 7 | 5 | 19.71429 | 0.761111 | 3704 | 0.425325 |
| HIF-2 signaling pathway | Pathway | 2.522222 | 0.010427 | 0.396476 | 0 | 6 | 5 | 14.5 | 0.746296 | 2312 | 0.321429 |
| TNF signaling pathway | Pathway | 2.388889 | 0.015638 | 0.418605 | 0 | 6 | 4 | 18.5 | 0.768519 | 3092 | 0.397727 |
| Neurotrophin signaling pathway | Pathway | 2.522222 | 0.005071 | 0.396476 | 0 | 6 | 5 | 19.16667 | 0.746296 | 1928 | 0.443089 |
| NF-kappaB signaling pathway | Pathway | 2.677778 | 0.009621 | 0.373444 | 0 | 5 | 5 | 13.6 | 0.72037 | 1536 | 0.35 |
| PI3K-Akt signaling pathway | Pathway | 2.522222 | 0.028793 | 0.396476 | 0 | 9 | 5 | 11.22222 | 0.746296 | 4866 | 0.269006 |
| NOD-like receptor signaling pathway | Pathway | 3.011111 | 0.002024 | 0.332103 | 0 | 4 | 5 | 16.25 | 0.664815 | 516 | 0.476563 |
| Toll-like receptor signaling pathway | Pathway | 2.5 | 0.003985 | 0.4 | 0 | 5 | 5 | 22.2 | 0.75 | 1882 | 0.493023 |
| B cell receptor signaling pathway | Pathway | 2.588889 | 0.002609 | 0.386266 | 0 | 4 | 5 | 19.75 | 0.735185 | 1024 | 0.46875 |
| MAPK signaling pathway | Pathway | 2.588889 | 0.010698 | 0.386266 | 0 | 7 | 5 | 15 | 0.735185 | 2506 | 0.341463 |
| Wnt signaling pathway | Pathway | 2.833333 | 0.024031 | 0.352941 | 0 | 5 | 5 | 12.6 | 0.694444 | 4346 | 0.351515 |
| Rap2 signaling pathway | Pathway | 2.588889 | 0.011398 | 0.386266 | 0 | 6 | 5 | 12.83333 | 0.735185 | 1830 | 0.303419 |
| ErbB signaling pathway | Pathway | 2.655556 | 0.002412 | 0.376569 | 0 | 4 | 5 | 19.25 | 0.724074 | 776 | 0.480263 |
| Chemokine signaling pathway | Pathway | 2.633333 | 0.016303 | 0.379747 | 0 | 5 | 5 | 14.2 | 0.727778 | 1932 | 0.347368 |
| NOS1 | Target | 3.088889 | 0.001395 | 0.323741 | 0 | 2 | 5 | 10.5 | 0.651852 | 280 | 0.527778 |
| SPHK1 | Target | 2.933333 | 0.002821 | 0.340909 | 0 | 3 | 4 | 10 | 0.677778 | 606 | 0.409091 |
| EDNRA | Target | 2.822222 | 0.00378 | 0.354331 | 0 | 3 | 4 | 13.66667 | 0.696296 | 926 | 0.469136 |
| EDNRB | Target | 2.822222 | 0.00378 | 0.354331 | 0 | 3 | 4 | 13.66667 | 0.696296 | 926 | 0.469136 |
| ADRB2 | Target | 3.111111 | 0.005338 | 0.321429 | 0 | 3 | 5 | 10 | 0.648148 | 400 | 0.473684 |
| CHRM3 | Target | 3.133333 | 0.001767 | 0.319149 | 0 | 2 | 5 | 11.5 | 0.644444 | 190 | 0.552632 |
| CHRM2 | Target | 3 | 0.003921 | 0.333333 | 0 | 3 | 5 | 10.66667 | 0.666667 | 588 | 0.42029 |
| ATP2A1 | Target | 3.222222 | 3.75E-04 | 0.310345 | 0 | 2 | 5 | 12 | 0.62963 | 84 | 0.6875 |
| ADRA1B | Target | 3.222222 | 3.75E-04 | 0.310345 | 0 | 2 | 5 | 12 | 0.62963 | 84 | 0.6875 |
| CHRNA7 | Target | 3.022222 | 0.021159 | 0.330882 | 0 | 3 | 5 | 9.333333 | 0.662963 | 1054 | 0.362319 |
| NOS3 | Target | 2.822222 | 0.006966 | 0.354331 | 0 | 5 | 4 | 9.4 | 0.696296 | 1250 | 0.336 |
| NOS2 | Target | 2.555556 | 0.031407 | 0.391304 | 0 | 10 | 4 | 8.4 | 0.740741 | 5828 | 0.23125 |
| PPP3CA | Target | 2.555556 | 0.044546 | 0.391304 | 0 | 12 | 4 | 7.25 | 0.740741 | 7542 | 0.208333 |
| F2R | Target | 2.755556 | 0.006442 | 0.362903 | 0 | 4 | 4 | 11.75 | 0.707407 | 1350 | 0.37069 |
| HTR2A | Target | 3.177778 | 0.001357 | 0.314685 | 0 | 2 | 5 | 9.5 | 0.637037 | 144 | 0.5 |
| PIK3CG | Target | 1.911111 | 0.268173 | 0.523256 | 0 | 32 | 4 | 6.5625 | 0.848148 | 35208 | 0.142628 |
| PTGS2 | Target | 2.622222 | 0.080618 | 0.381356 | 0 | 8 | 4 | 7.375 | 0.72963 | 7376 | 0.205645 |
| ADCY5 | Target | 2.577778 | 0.027077 | 0.387931 | 0 | 8 | 4 | 7.875 | 0.737037 | 3450 | 0.208333 |
| PPARG | Target | 3 | 4.51E-04 | 0.333333 | 0 | 2 | 4 | 12 | 0.666667 | 72 | 0.55 |
| TP53 | Target | 2.555556 | 0.036595 | 0.391304 | 0 | 15 | 4 | 6.933333 | 0.740741 | 6342 | 0.219753 |
| CXCL8 | Target | 2.622222 | 0.038363 | 0.381356 | 0 | 15 | 4 | 6.4 | 0.72963 | 7632 | 0.225 |
| SMAD3 | Target | 2.911111 | 0.005116 | 0.343511 | 0 | 6 | 4 | 7.833333 | 0.681481 | 1160 | 0.341667 |
| BIRC2 | Target | 2.888889 | 0.007607 | 0.346154 | 0 | 7 | 4 | 7.285714 | 0.685185 | 1618 | 0.314286 |
| MMP1 | Target | 3.044444 | 9.37E-04 | 0.328467 | 0 | 2 | 4 | 11 | 0.659259 | 284 | 0.555556 |
| JUN | Target | 2.511111 | 0.06056 | 0.39823 | 0 | 21 | 4 | 6.52381 | 0.748148 | 12198 | 0.240166 |
| BCL2 | Target | 2.488889 | 0.04437 | 0.401786 | 0 | 14 | 4 | 7.357143 | 0.751852 | 7494 | 0.205069 |
| GSK3B | Target | 2.555556 | 0.03015 | 0.391304 | 0 | 14 | 4 | 7.142857 | 0.740741 | 5376 | 0.219388 |
| EGF | Target | 2.688889 | 0.018799 | 0.371901 | 0 | 10 | 4 | 7 | 0.718519 | 3426 | 0.230769 |
| MAPK14 | Target | 2.488889 | 0.055351 | 0.401786 | 0 | 19 | 4 | 6.526316 | 0.751852 | 9548 | 0.212551 |
| HSPB1 | Target | 3.044444 | 0.001578 | 0.328467 | 0 | 4 | 4 | 7.25 | 0.659259 | 308 | 0.390625 |
| KDR | Target | 3 | 0.003364 | 0.333333 | 0 | 4 | 4 | 7.25 | 0.666667 | 712 | 0.347222 |
| Chagas disease (American trypanosomiasis) | Target | 2.411111 | 0.012907 | 0.414747 | 0 | 8 | 5 | 17.875 | 0.764815 | 4118 | 0.383523 |
| TNF | Target | 2.4 | 0.085235 | 0.416667 | 0 | 24 | 4 | 6 | 0.766667 | 16280 | 0.2 |
| IFNG | Target | 2.622222 | 0.035183 | 0.381356 | 0 | 16 | 4 | 6.125 | 0.72963 | 7448 | 0.222826 |
| Cholinergic synapse | Target | 2.544444 | 0.07692 | 0.393013 | 0 | 8 | 5 | 8 | 0.742593 | 8840 | 0.194444 |
| ACHE | Target | 3.533333 | 0 | 0.283019 | 0 | 1 | 6 | 8 | 0.577778 | 0 | 0 |
| CHRNA4 | Target | 3.533333 | 0 | 0.283019 | 0 | 1 | 6 | 8 | 0.577778 | 0 | 0 |
| Amyotrophic lateral sclerosis (ALS) | Target | 2.566667 | 0.016835 | 0.38961 | 0 | 6 | 5 | 14.33333 | 0.738889 | 2986 | 0.325203 |
| Toxoplasmosis | Target | 2.455556 | 0.032147 | 0.40724 | 0 | 8 | 5 | 15.375 | 0.757407 | 4972 | 0.334302 |
| ALOX5 | Target | 3.444444 | 0 | 0.290323 | 0 | 1 | 6 | 8 | 0.592593 | 0 | 0 |
| Regulation of lipolysis in adipocytes | Target | 2.611111 | 0.055497 | 0.382979 | 0 | 6 | 5 | 8.833333 | 0.731481 | 5948 | 0.22381 |
| PTGS1 | Target | 3.6 | 0 | 0.277778 | 0 | 1 | 6 | 6 | 0.566667 | 0 | 0 |
| FABP4 | Target | 3.6 | 0 | 0.277778 | 0 | 1 | 6 | 6 | 0.566667 | 0 | 0 |
| Hepatitis C | Target | 2.433333 | 0.030891 | 0.410959 | 0 | 8 | 5 | 16.25 | 0.761111 | 4420 | 0.354651 |
| SCARB1 | Target | 3.422222 | 0 | 0.292208 | 0 | 1 | 6 | 8 | 0.596296 | 0 | 0 |
| Colorectal cancer | Target | 2.544444 | 0.005898 | 0.393013 | 0 | 6 | 5 | 17 | 0.742593 | 1636 | 0.4 |
| Leishmaniasis | Target | 2.544444 | 0.014324 | 0.393013 | 0 | 6 | 5 | 16.33333 | 0.742593 | 2492 | 0.393162 |
| Influenza A | Target | 2.433333 | 0.029912 | 0.410959 | 0 | 8 | 5 | 17.75 | 0.761111 | 4608 | 0.389535 |
| PRSS1 | Target | 3.422222 | 0 | 0.292208 | 0 | 1 | 6 | 8 | 0.596296 | 0 | 0 |
| Small cell lung cancer | Target | 2.455556 | 0.012785 | 0.40724 | 0 | 6 | 4 | 14.33333 | 0.757407 | 2144 | 0.325203 |
| Osteoclast differentiation | Target | 2.433333 | 0.014381 | 0.410959 | 0 | 7 | 5 | 18 | 0.761111 | 3428 | 0.386364 |
| Epstein-Barr virus infection | Target | 2.433333 | 0.014247 | 0.410959 | 0 | 8 | 5 | 16.875 | 0.761111 | 3562 | 0.369186 |
| Apoptosis | Target | 2.522222 | 0.004996 | 0.396476 | 0 | 5 | 5 | 18.4 | 0.746296 | 1576 | 0.414286 |
| Amoebiasis | Target | 2.5 | 0.009892 | 0.4 | 0 | 6 | 5 | 16.83333 | 0.75 | 2458 | 0.368217 |
| Tuberculosis | Target | 2.588889 | 0.014219 | 0.386266 | 0 | 7 | 5 | 14 | 0.735185 | 3086 | 0.333333 |
| Chemical carcinogenesis | Target | 3.3 | 0.067437 | 0.30303 | 0 | 5 | 5 | 2.8 | 0.616667 | 4566 | 0.2 |
| CYP3A4 | Target | 4.288889 | 0 | 0.233161 | 0 | 1 | 6 | 5 | 0.451852 | 0 | 0 |
| CBR1 | Target | 4.288889 | 0 | 0.233161 | 0 | 1 | 6 | 5 | 0.451852 | 0 | 0 |
| CYP2A6 | Target | 4.288889 | 0 | 0.233161 | 0 | 1 | 6 | 5 | 0.451852 | 0 | 0 |
| Salmonella infection | Target | 2.722222 | 0.003269 | 0.367347 | 0 | 5 | 5 | 16.2 | 0.712963 | 1182 | 0.410811 |
| HTLV-I infection | Target | 2.411111 | 0.015272 | 0.414747 | 0 | 8 | 5 | 16.5 | 0.764815 | 4290 | 0.352273 |
| Rheumatoid arthritis | Target | 2.966667 | 0.006001 | 0.337079 | 0 | 5 | 5 | 15.6 | 0.672222 | 852 | 0.442424 |
| Hepatitis B | Target | 2.477778 | 0.006478 | 0.403587 | 0 | 6 | 5 | 20.16667 | 0.753704 | 2658 | 0.445736 |
| Inflammatory bowel disease (IBD) | Target | 2.988889 | 0.002117 | 0.334572 | 0 | 4 | 5 | 16.75 | 0.668519 | 484 | 0.477273 |
| Pancreatic cancer | Target | 2.677778 | 0.002967 | 0.373444 | 0 | 4 | 5 | 15.75 | 0.72037 | 614 | 0.409722 |
| SFRP1 | Target | 3.822222 | 0 | 0.261628 | 0 | 1 | 6 | 5 | 0.52963 | 0 | 0 |
| Non-alcoholic fatty liver disease (NAFLD) | Target | 2.5 | 0.004062 | 0.4 | 0 | 5 | 5 | 21.2 | 0.75 | 1798 | 0.469767 |
| Cytokine-cytokine receptor interaction | Target | 2.922222 | 0.015815 | 0.342205 | 0 | 6 | 5 | 11.83333 | 0.67963 | 1734 | 0.318627 |
| CCR6 | Target | 3.377778 | 3.74E-04 | 0.296053 | 0 | 2 | 6 | 5.5 | 0.603704 | 116 | 0.5625 |
| Inflammatory mediator regulation of TRP channels | Target | 2.655556 | 0.01348 | 0.376569 | 0 | 4 | 5 | 15.25 | 0.724074 | 1972 | 0.375 |
| Malaria | Target | 3.144444 | 4.93E-04 | 0.318021 | 0 | 3 | 5 | 18.33333 | 0.642593 | 184 | 0.619048 |
| Non-small cell lung cancer | Target | 2.722222 | 0.00126 | 0.367347 | 0 | 3 | 5 | 19 | 0.712963 | 348 | 0.514286 |
| Natural killer cell mediated cytotoxicity | Target | 2.5 | 0.003721 | 0.4 | 0 | 4 | 5 | 21 | 0.75 | 1622 | 0.454545 |
