# Supplementary material for: Systems Pharmacology and Verification of ShenFuHuang Formula in Zebrafish Model Reveal Multi-Scale Treatment Strategy for Septic Syndrome in COVID-19
Source: Front Pharmacol. 2020 Sep 15;11:584057. doi: 10.3389/fphar.2020.584057 (PMC7523021; doi:10.3389/fphar.2020.584057)
Supplement: Supplementary file 5 [file Table_5.docx]

| Name | ID | Content (μg/mL) |
| --- | --- | --- |
| Aloe-emodin | Sample 1  Sample2  Sample3 | 50.4632  48.1584  45.772 |
| Rhein | Sample 1  Sample2  Sample3 | 93.816  96.584  141.692 |
| Fuziline | Sample 1  Sample2  Sample3 | 80.212  61.3048  64.8384 |
| Deoxyaconitine | Sample 1  Sample2  Sample3 | 13.0504  11.7168  11.4816 |
| Ginsenoside rh2 | Sample 1  Sample2  Sample3 | 1.3784  1.5368  2.3192 |
| Quercetin | Sample 1  Sample2  Sample3 | 2.8776  1.6304  1.5288 |
| Gallic acid | Sample 1  Sample2  Sample3 | 159.3208  155.736  157.356 |

**Supplementary Table S5**

**Content of dominating compounds**

Ion chromatogram of SFH Formula (3 samples)

**SFH formula, Sample1, Positive ion flow**

**SFH formula, Sample2, Positive ion flow**

**SFH formula, Sample3, Positive ion flow**

**SFH formula, Sample1, Negative ion flow**

**SFH1 formula, Sample2, Negative ion flow**

**SFH formula, Sample3, Negative ion flow**

**Chromatogram of dominating compounds**

**Aloe-emodin**

**Rhein**

**Fuziline**

**Deoxyaconitine**

**Ginsenoside rh2**

**Quercetin**

**Gallic acid**
